# NIMA-related kinase family at the nexus of skeletal development and congenital arthrogryposis: coordinated regulation of cell cycle and ciliary dynamics

**DOI:** 10.3389/fgene.2026.1883963

**Published:** 2026-07-13

**Authors:** Yitong Zhao, Ying Zheng, Qian Chen, Ying Yang, Liyu Zhang

**Affiliations:** 1 Shaanxi Institute for Pediatric Diseases, Xi’an Children’s Hospital, Xi’an, Shaanxi, China; 2 Key Laboratory for High Altitude Brain Injury and Repair, School of Medicine, Xizang Minzu University, Xianyang, Shaanxi, China

**Keywords:** cell cycle, ciliogenesis, congenital arthrogryposis, nek kinase family, skeletal development

## Abstract

**Background:**

The NIMA-related kinase (NEK) family comprises a group of serine/threonine kinases that play pivotal roles in cell cycle control. Emerging evidence has demonstrated that loss-of-function (LOF) mutations in NEK family members, particularly NEK1 and NEK9, cause skeletal and joint developmental abnormalities.

**Results:**

This review synthesizes clinical, genetic, and functional evidence from patient-based studies, cell models, and animal experiments to elucidate the pathogenic mechanisms. Loss-of-function of these two kinases can disrupt cell cycle progression through distinct mechanisms, such as arrest at different phases (e.g., S phase and G2/M phase). Although the arrest stages differ, both can be accompanied by defects in primary cilia formation, which in turn disrupt cilia-dependent Hedgehog and Wnt signaling. The interplay between cell cycle dysregulation and ciliary dysfunction is tightly intertwined in these diseases; however, it remains difficult to clearly distinguish cause from effect between the two. NEK kinases may drive pathology through multiple pathways, including directly affecting cilia assembly and interfering with cell cycle progression. This dual impairment of the cell cycle and cilia leads to chondrocyte proliferation failure, growth plate disorganization, and joint contractures. Genotype–phenotype correlations reveal that missense versus truncating NEK1 mutations cause divergent skeletal phenotypes, while NEK9 mutations underlie lethal congenital contracture syndrome. Emerging evidence also suggests potential modifying roles for NEK7 in inflammation and endosomal trafficking.

**Conclusion:**

In conclusion, NEK-associated skeletal disorders arise from a combination of cell cycle defects and ciliary abnormalities, rather than being caused by either factor alone. Consequently, therapeutic strategies targeting cell cycle regulation or related downstream pathways may offer new avenues for treating these severe pediatric skeletal diseases.

## Graphical Abstract

**Figure:**
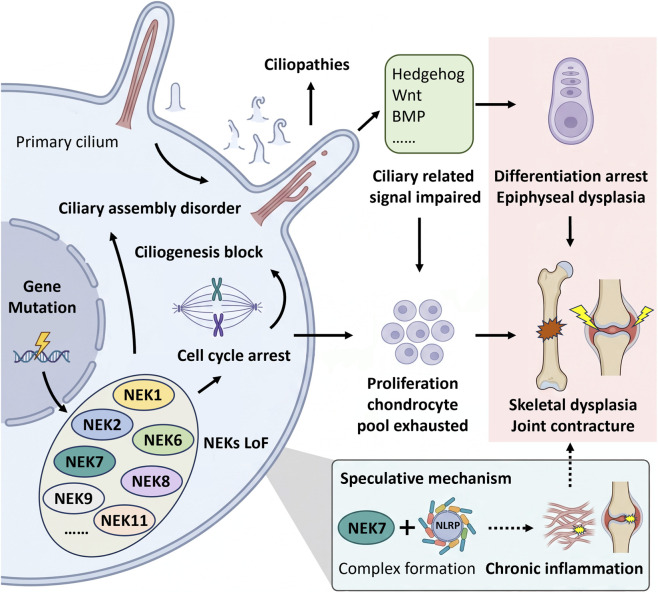
Loss-of-function (LOF) mutations in NEKs can induce cell cycle arrest, ciliary defects, and potentially chronic inflammation, ultimately leading to skeletal dysplasia. LOF mutations in NEK family kinase members, including but not limited to NEK1, NEK2, NEK6, NEK7, NEK8, NEK9, and NEK11, on one hand, induce cell cycle arrest and further inhibit ciliogenesis. On the other hand, loss of function of certain NEK family members also directly impairs cilia formation. Defects in primary cilia formation can lead to ciliopathies and disrupt signaling pathways such as Hedgehog, Wnt, and BMP, resulting in abnormal cell differentiation during bone formation. Meanwhile, cell cycle arrest caused by NEK deficiency directly inhibits chondrocyte proliferation, depletes the chondrocyte pool, and ultimately leads to epiphyseal dysplasia, skeletal deformities, and joint contractures. Furthermore, NEK7 can bind to the NLRP inflammasome complex and promote its assembly, triggering chronic inflammation, which may further exacerbate the pathological progression of skeletal dysplasia.

## 1 Introduction

Skeletal dysplasia (SD), also known as osteochondrodysplasia (OCD), encompasses a broad spectrum of disorders arising from aberrant skeletal development and growth, with an estimated overall incidence of approximately 1 in 5,000 live births (Billich et al., 2023; Handa et al., 2022; Jurca et al., 2021). According to the most recent classification criteria established by the International Skeletal Dysplasia Society (ISDS), this category comprises 461 distinct entities organized into 42 major groups, among which 437 causative genes have been identified and account for approximately 92.4% of disease subtypes (Jurca et al., 2021; Mortier et al., 2019; Stembalska et al., 2021). Radiological assessment, particularly plain radiography, remains the cornerstone of clinical diagnosis (Handa et al., 2022; Handa et al., 2023; Aparisi Gomez et al., 2021). Epidemiological evidence indicates that autosomal recessive mutations constitute the predominant pathogenic factor in specific populations, a phenomenon significantly associated with elevated rates of consanguineous marriage (Jacob et al., 2025). These disorders show a wide phenotypic spectrum, from mild non-lethal to severe perinatal lethal forms.

For instance, *TGDS* variants cause deficiency of UDP-4-keto-6-deoxyglucose, leading to UXS1 inactivation and impaired synthesis of glycan chains critical for skeletal development, thereby generating the diverse clinical phenotypes observed in Catel–Manzke syndrome (Jacobs et al., 2025). Similarly, distinct *SLC26A2* variants can result in a phenotypic continuum spanning from relatively mild multiple epiphyseal dysplasia to lethal achondrogenesis (Harkonen et al., 2021). Patients harboring biallelic *KIF24* variants may also present with a clinical spectrum extending from severe acromesomelic dysplasia to lethal skeletal ciliopathy (Reilly et al., 2022). These observations underscore the complexity of genotype-phenotype correlations in skeletal system development. Notably, such complexity may manifest during intrauterine development through restriction of fetal movement, ultimately culminating in the phenotypic outcome of joint contractures. Arthrogryposis multiplex congenita (AMC), as a rare skeletal disorder, represents a constellation of multiple joint contractures and skeletal deformities resulting from diverse etiologies including skeletal dysplasia, rather than a single nosological entity (Langston and Chu, 2020; Khurana et al., 2024; Ma and Yu, 2017).

Ciliopathies with major skeletal involvement comprise a group of autosomal recessive disorders caused by pathogenic variants in multiple genes encoding components of the primary cilium, and characterized predominantly by marked alterations in skeletal architecture (Handa et al., 2020; Peng et al., 2023; Shamseldin et al., 2020). Recent studies have substantially expanded the spectrum of causative genes associated with this disease category. For example, variants in *NEK1*, *CEP120*, *TCTN3*, *GRK2*, and *KIAA0753* disrupt primary ciliary function, resulting in clinical phenotypes featuring narrow thorax, shortened long bones, hypotonia, developmental delay, and respiratory and feeding difficulties—manifestations highly concordant with Jeune syndrome (Faudi et al., 2020; Topcu et al., 2024). Loss of Sprouty family proteins induces ciliopathy-like skeletal dysplasia in mice through upregulation of Hedgehog signaling (Hruba et al., 2021). Deficiency of *TTC30A/B* impairs ciliogenesis secondary to altered posttranslational tubulin acetylation and glycylation, together with defective axoneme compartmentalization, establishing these proteins as essential nodes within the regulatory network of ciliary chondrodysplasia and nephronophthisis-associated disease proteins. Moreover, accumulating evidence indicates that tubulin modifications and ciliary compartmentalization contribute to the skeletal and renal manifestations of ciliopathies in a cell type-specific manner (Getwan et al., 2021).

Within this spectrum of cilia-associated skeletal disorders, the NEK kinase family has emerged as critical pathogenic molecules. Pathogenic mutations in *NEK1* and *NEK9* have been definitively identified as causative for severe lethal skeletal dysplasia and congenital arthrogryposis syndromes, which are mediated by mechanisms involving cell cycle arrest and defective ciliogenesis (Casey et al., 2016; Thiel et al., 2011). *NEK1* has been incorporated into the curated gene list for skeletal ciliopathies by multiple independent studies. The OMIM database catalogs *NEK1* mutations as causative of Majewski-type short-rib thoracic dysplasia (SRTD), with pathogenesis intimately linked to aberrant ciliary assembly (Thiel et al., 2011; Chen et al., 2018; Gregorczyk et al., 2023; El Hokayem et al., 2012). Mutations in *NEK9* underlie lethal congenital contracture syndrome 10 (LCCS10; OMIM 617022). The index patient harboring homozygous nonsense mutation in *NEK9* c.1489C>T (p.Arg497*) exhibited cell cycle progression arrest in fibroblasts, concomitant with marked reduction in both ciliary number and length (Casey et al., 2016).

Additionally, NEK9 has been implicated in organizing spindle formation during mitosis, regulating cell cycle progression, and coordinating cytokinesis (Kaneta and Ullrich, 2013). Notably, these two cellular phenotypes—cell cycle arrest and ciliary abnormalities—are not merely parallel pathological events but are tightly intertwined and mutually interactive: Loss-of-function (LOF) of NEK2, NEK6, NEK7, and NEK9, which function as essential cell cycle regulators, can lead to mitotic arrest, particularly defective G2/M transition, trapping cells in the active cell cycle and preventing their exit into quiescence (G0 phase), thereby severely impairing primary cilium assembly (Fry et al., 2012; Panchal and Evan, 2023). However, NEK kinases may also play a direct regulatory role in cilia assembly and maintenance; therefore, the causal relationship between cell cycle dysregulation and ciliary dysfunction in these diseases may not be a unidirectional linear chain but rather a bidirectional interactive network (Zhao et al., 2021; De Jesus-Rojas et al., 2023; Kim and Lee, 2017).

This review focuses on the NEK kinase family, with particular emphasis on NEK1 and NEK9. We systematically summarize the clinical manifestations, genetic basis, and cellular and molecular mechanisms underlying their roles as key pathogenic genes in skeletal developmental disorders. Furthermore, we explore how the NEK kinase family integrates cell cycle and ciliogenesis-related signaling to participate in the common regulatory network of skeletal system development. These discussions aim to provide a novel theoretical framework for advancing research in this field and facilitating clinical translation.

## 2 The NEK kinase family: overview and their core cell biological functions

### 2.1 Evolutionary conservation, classification, and structural characteristics

The NEK (NIMA-related kinase) family comprises a group of evolutionarily conserved serine/threonine kinases. Their kinase domains share homology with the NIMA (Never In Mitosis A) kinase originally identified in filamentous fungi such as *Aspergillus nidulans* (Bachus et al., 2022; Li H. et al., 2025). The human NEK family contains eleven members (NEK1–NEK11) that participate broadly in critical cellular processes including cell cycle regulation, spindle assembly, DNA damage response, and ciliary biology (Fry et al., 2012; Panchal and Evan, 2023; Quarmby and Mahjoub, 2005; O'Regan et al., 2007; Baker et al., 2025). From fungi to mammals, NIMA-related kinases exhibit strong evolutionary conservation in the core sequences of their kinase domains. They are also involved to varying degrees in the regulation of microtubule-dependent processes. These observations suggest an indispensable role for this kinase family in maintaining fundamental cellular activities (Fry et al., 2012; Baker et al., 2025).

NEK family members localize to distinct cellular compartments and can be further classified into subgroups based on sequence similarity, structural features, and functional characteristics (Panchal and Evan, 2023; Baker et al., 2025). For instance, NEK2, NEK6, NEK7, and NEK9 primarily participate in the establishment and regulation of the mitotic spindle, whereas NEK1, NEK10, and NEK11 are more extensively involved in DNA damage response pathways (Fry et al., 2012; Quarmby and Mahjoub, 2005; Baker et al., 2025). Systematic analysis of substrate specificity across the NEK kinase family has revealed that all members prefer substrates with hydrophobic amino acids at the −3 position. However, they exhibit differential preferences for serine versus threonine phosphorylation acceptors, as well as selective specificity for basic or acidic residues at other positions (van de Kooij et al., 2019). Notably, NEK10 functions as a dual-specificity kinase that efficiently phosphorylates both serine and tyrosine residues within itself and peptide substrates. Its activity can be enhanced through autophosphorylation at tyrosine sites, thereby displaying characteristics distinct from other family members (van de Kooij et al., 2019).

Structurally, canonical NEK kinases comprise an N-terminal catalytic kinase domain and a C-terminal regulatory domain. Extensive available data have elaborated the differences in domain organization among family members (Fry et al., 2012; Baker et al., 2025). The N-terminal catalytic domain represents a conserved sequence shared across the family and contains all typical motifs of serine/threonine kinases. All eleven human NEKs harbor the His-Arg-Asp (HRD) motif within their catalytic domains. They all contain serine or threonine residues within their activation loops, which serve as potential sites for activating modifications (Fry et al., 2012; Baker et al., 2025; Johnson et al., 1996). In contrast, the C-terminal regulatory domains vary considerably in length, sequence, and domain organization. These domains play critical roles in mediating protein interactions, determining subcellular localization, and regulating kinase activity. They constitute the core of functional diversity and specific regulatory mechanisms within the NEK family.

For example, NEK2A is the major splice variant (isoform) encoded by the NEK2 gene and plays a central role in the regulation of mitosis. NEK2A localizes to the nucleolus through a specific C-terminal region (amino acids 399–445). NEK11 interacts with the non-catalytic region of NEK2A and becomes activated through phosphorylation by the latter (Noguchi et al., 2004). PLK1 serves as a direct upstream activator of NEK9, while NEK9 itself undergoes autophosphorylation in its non-catalytic C-terminal region to regulate its subcellular localization and activation status (Bertran et al., 2011). Such modular architecture enables different NEK members to integrate and respond to diverse upstream signals. Through phosphorylating specific substrates, they precisely regulate biological events including cell cycle progression, genome stability maintenance, and ciliary assembly (Fry et al., 2012; Bachus et al., 2022). Therefore, comprehensive analysis of the structure, classification, and evolutionary relationships of the NEK family constitutes an essential prerequisite for elucidating their specific mechanistic roles in skeletal development and disease.

### 2.2 The core regulatory roles in cell cycle progression

**FIGURE 1:**
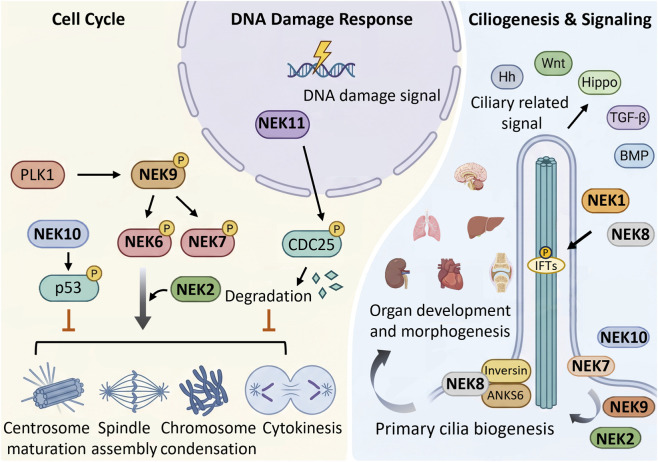
Regulatory network of NEK family kinases in cell cycle progression, DNA damage response, ciliogenesis, and associated signaling pathways. NEK family members form a molecular regulatory network with key regulators such as PLK1, CDC25, p53, IFTs, Inversin, and ANKS6, coordinating centrosome function, spindle assembly, chromosome condensation, cytokinesis, and primary cilia biogenesis and maturation. These processes integrate cilia-related signals (e.g., Wnt, Hedgehog, Hippo, TGF-β, BMP) and DNA damage signals, thereby regulating tissue and organ development and morphogenesis.

The NEK kinase family functions as serine/threonine kinases that play central roles in cell cycle regulation, particularly during mitotic progression (Figure 1; Supplementary Table S1) (Fry et al., 2012; Folahan and Barabutis, 2025). Most members of the NEK family play essential roles in cell cycle regulation, including checkpoint control, mitotic progression, spindle assembly, and cytokinesis, underscoring their broad but non-redundant functions in ensuring faithful cell division. Among these, NEK2, NEK6, NEK7, and NEK9 have been established as critical regulators of mitosis. They participate extensively in a series of precise processes including centrosome maturation and separation, spindle assembly, chromosome condensation, and cytokinesis (Fry et al., 2012; O'Regan et al., 2007; Cullati et al., 2017). The functions of these kinases are not independent but rather form a complex regulatory network with functional interconnections. NEK9 serves as an upstream activator that becomes phosphorylated and activated by PLK1 upon mitotic entry. It subsequently transmits signals through two independent pathways to phosphorylate and activate its downstream effector kinases NEK6 and NEK7, thereby regulating distinct aspects of cytokinesis (Bertran et al., 2011; Belham et al., 2003).

Studies have demonstrated that NEK2 expression is significantly upregulated in hepatocellular carcinoma. Inhibition of NEK2 suppresses cancer cell proliferation, growth, and colony formation, and induces G2/M phase cell cycle arrest and apoptosis (Lai et al., 2017). NEK6 is highly expressed in osteosarcoma and other tumors, where it promotes cell cycle progression and metastasis through regulating STAT3 signaling pathways (Zhu et al., 2023). NEK7 is aberrantly overexpressed in various tumors and facilitates cell proliferation via modulating cell cycle checkpoints (Zhang et al., 2018; Li YK. et al., 2021). NEK10 has been shown to directly phosphorylate the tyrosine residue (Y327) of the tumor suppressor p53, thereby inhibiting p53 transcriptional activity and cell proliferation (Haider et al., 2020).

NEK11 represents another important family member whose functions are intimately linked to DNA damage response and cell cycle checkpoint control. NEK11 plays a critical role in the DNA damage-induced G2/M checkpoint. It achieves this by phosphorylating and promoting the degradation of CDC25A, thereby preventing cell cycle entry into mitosis (Sorensen et al., 2010; Melixetian et al., 2009). The precise regulation of key mitotic events by the NEK family is essential for maintaining genomic stability. Dysfunction of these kinases can lead to centrosome abnormalities, spindle assembly defects, and chromosomal instability. Such functional impairments render the NEK family promising targets for novel chemotherapeutic strategies (O'Regan et al., 2007; Marzin and Cormier-Daire, 2020).

This precise mitotic regulatory mechanism holds particular significance for skeletal system development, especially for longitudinal bone growth. Chondrocytes in the growth plate must undergo rapid yet orderly proliferation, differentiation, and apoptosis to drive linear skeletal extension, and their proliferative efficiency directly determines the rate of bone growth (Rosello-Diez and Joyner, 2015; Putz and Milz, 2016; Nilsson and Baron, 2004; Zhao et al., 2022). Abnormalities in the expression or activity of this family may disrupt the proliferative rhythm of chondrocytes and impair normal skeletal development. Such dysregulation may represent one of the potential molecular mechanisms underlying certain skeletal developmental disorders or congenital arthrogryposis syndromes.

### 2.3 Multifunctionality roles in ciliogenesis and signal transduction

The functional repertoire of the NEK kinase family extends well beyond cell cycle regulation, with several members playing central roles in the biogenesis of primary cilia (Bachus et al., 2022; Quarmby and Mahjoub, 2005; Baker et al., 2025). The influence of NEK kinases on ciliary signaling capacity is achieved primarily through precise regulation of the intraflagellar transport (IFT) machinery (Figure 1; Supplementary Table S1) (Baker et al., 2025; Lacigova and Cajanek, 2025). IFT depends on a bidirectional transport system powered by kinesin and dynein motors, which is responsible for trafficking ciliary structural components and signaling molecules between the ciliary base and tip. NEK kinases serve as molecular switches for this process through phosphorylating key components of the IFT motor proteins (Chen G. et al., 2025).

Primary cilia are microtubule-based non-motile organelles whose assembly, maintenance, and disassembly are tightly coupled to the cell cycle. They typically form when cells exit the cell cycle and enter quiescence (G0 phase), and both NEK1 and NEK8 participate in regulating this process (Fry et al., 2012). Studies have shown that loss of NEK1 function severely reduces ciliary number and alters ciliary morphology (Thiel et al., 2011). Mutations in NEK1 have been confirmed in association with short-rib thoracic dysplasia type 6 (SRTD6) and other skeletal ciliopathies, providing direct evidence for its involvement in skeletal development through ciliary pathways (Thiel et al., 2011; Gregorczyk et al., 2023; El Hokayem et al., 2012).

NEK7 regulates dendritic morphogenesis, and its kinase activity is essential for neuronal dendritic growth and branching as well as spine formation and morphological development (Freixo et al., 2018). NEK8 functions as a component of the INV ciliary compartment core complex, where it interacts with various cilia-associated proteins including inversin, ANKS6, and NPHP3. Its correct localization to the proximal cilium is critical for ciliary signaling function and proper development of organs such as the kidney, heart, and liver (Roig, 2025). NEK10 has been established as a ciliated cell-specific kinase that regulates ciliary growth and mucociliary transport in airway epithelia (Chivukula et al., 2020).

Cilia are not static structures; rather, they serve as cellular “signaling antennae” responsible for transducing key signaling pathways, such as Hedgehog, Wnt, Hippo, TGF-β, and BMP signaling that are critical for embryonic development and tissue homeostasis, among which with the Hedgehog pathway is the most extensively characterized (Song et al., 2026; Avruch et al., 2012; Li B. et al., 2025; Anvarian et al., 2019; Ishikawa and Marshall, 2011). These signaling pathways are indispensable for pattern formation in the skeletal system, endochondral ossification, and joint development (Costantini et al., 2023; Guasto and Cormier-Daire, 2021). As signaling hubs for skeletogenesis, precise regulation of ciliary biogenesis constitutes the core molecular basis for understanding how NEK family mutations cause developmental disorders characterized by skeletal and joint malformations, such as congenital arthrogryposis syndromes.

NEK kinases indirectly yet profoundly influence the activity of these skeletal developmental signaling pathways through regulating ciliary integrity. NEK1 and NEK8 localize to cilia and coordinate microtubule structural formation. Their functional impairment leads to ciliary defects and dysfunction, which may subsequently contribute to skeletal developmental disorders through the aforementioned signaling pathways, and this may further exacerbate cilia-associated abnormal phenotypes caused by NEK9 deficiency (Thiel et al., 2011; Fry et al., 2012; Roig, 2025).

These findings extend the functional repertoire of NEK kinases from cell cycle regulation to ciliary regulation, microtubule dynamics, cell growth, and DNA damage response. They suggest that this family coordinates microtubule-dependent processes to exert important roles in both dividing and non-dividing cells, thereby exerting impact across broader physiological and pathological contexts.

## 3 NEK1 and NEK9: central drivers of skeletal dysplasia and arthrogryposis

### 3.1 Phenotypic and genotypic landscape of NEK1 variants

NEK1 functions as a multifunctional serine/threonine kinase that plays central roles in maintaining genomic stability, ciliary function, and cell cycle regulation. LOF mutations in this gene are closely associated with a spectrum of autosomal recessive disorders, and the severity of clinical phenotypes correlates clearly with mutation types (Supplementary Table S2) (Thiel et al., 2011; Chen et al., 2018; Gregorczyk et al., 2023; El Hokayem et al., 2012; Kenna et al., 2016; Jiang et al., 2023; Watanabe et al., 2025). For example, mutations at specific loci such as c.379C>T (p.Arg127*) and c.869-2A>G in the *NEK1* gene could cause Short-Rib Polydactyly Syndrome type II (SRPII, Majewski type). These mutations lead to severe defects in ciliary structure and function, thereby affecting skeletal development and viability. Affected patients present with short ribs, short limbs, polydactyly, and multiple major organ anomalies (Thiel et al., 2011). This condition represents a lethal disorder, with death typically occurring in the perinatal or neonatal period (El Hokayem et al., 2012).

It has been reported that one patient with Short-Rib Thoracic Dysplasia type 6 (SRTD6) who had been misdiagnosed for an extended period was ultimately confirmed to harbor a homozygous nonsense pathogenic variant in *NEK1* c.1957C>T (p.R653*) (Zhang et al., 2025). This patient exhibited major features including severe rib and sternal hypoplasia. Notably, the mutation spectrum of *NEK1* demonstrates significant genetic heterogeneity. Compound heterozygous mutations c.3107C>G (p.S1036*) and c.3830A>C (p.D1277A) were identified in a patient with axial spondylometaphyseal dysplasia (axial SMD) (Wang et al., 2017). The skeletal phenotype in this case was milder than previously reported cases harboring *NEK1* mutations or *C21orf2* mutations associated with axial SMD, although the patient presented with retinal dystrophy, which expands the phenotypic spectrum of NEK1-related skeletal ciliopathies (Wang et al., 2017). Similar ciliopathy-associated features have been observed in mouse models harboring homozygous mutations in *Nek1* (Upadhya et al., 2000). The aforementioned genotype-phenotype correlations suggest that the presence or absence of residual NEK1 protein activity may partially determine disease severity.

Furthermore, *NEK1* serves as a susceptibility gene for amyotrophic lateral sclerosis (ALS), extending its functional relevance beyond skeletal development to encompass broader cellular homeostasis maintenance (Jiang et al., 2023; Noh et al., 2025; Rifai et al., 2025). Large-scale genetic studies have identified LOF variants in *NEK1* in both sporadic and familial ALS patients (Jiang et al., 2023; Pensato et al., 2025). The detection rate of rare *NEK1* variants in Italian ALS patients is approximately 2.85%. Some of these patients concurrently carry mutations in other major ALS causative genes such as *C9orf72* and *SOD1*, suggesting an oligogenic inheritance pattern for ALS (Pensato et al., 2025). One study performed whole-exome sequencing on 1,587 Chinese patients with ALS and identified NEK1 variants in 42 cases (2.6%). Among these, patients carrying loss-of-function variants had a significantly shorter median survival time (23.80 months) compared to those carrying pathogenic missense variants (42.77 months). (Jiang et al., 2023).

Dysregulation of NEK1-dependent DNA damage repair and neuronal survival represents one of the core mechanisms underlying ALS pathogenesis. Functional studies have demonstrated that LOF mutations in *NEK1* lead to increased accumulation of DNA damage in patient-derived iPSC motor neurons, accompanied by impaired repair capacity following damage (Higelin et al., 2018). Additionally, *NEK1* variants can cause primary ciliary dysfunction, cell cycle re-entry arrest, and tubulin acetylation disturbances. These abnormalities subsequently contribute to ALS pathogenesis through disrupting neuronal homeostasis (Noh et al., 2025). Neuropathological analysis has confirmed that ALS cases harboring LOF mutations in NEK1 (p.Arg261His) exhibit pathological cytoplasmic aggregation of TDP-43. This finding provides novel insights into the pathological characteristics of NEK1-associated ALS (Rifai et al., 2025).

### 3.2 Clinical recognition and phenotypic spectrum of NEK9 variants

At present, pathogenic variants in the NEK9 gene are closely associated with severe skeletal developmental disorders and arthrogrypotic phenotypes (Supplementary Table S2) (Casey et al., 2016). NEK9 has been established as a critical regulator of primary cilium formation, and its functional loss disrupts ciliogenesis (Yamamoto et al., 2021). The NEK9 mutation c.1489C>T(p.Arg497*) was initially reported in two Irish Traveler families. Affected individuals presented with severe skeletal dysplasia characterized by shortened long bones, multiple contractures, and rib and thoracic vertebral anomalies, together with reduced cell proliferation, delayed cell cycle progression, and ciliary defects (Casey et al., 2016). A case study of neonatal arthrogryposis syndrome reported the association of compound heterozygous variants in NEK9 with neonatal joint contractures through family-based whole exome sequencing (Liu et al., 2022). The two patients in this study carried compound heterozygous variants c.717C>A (p.C239*741) and c.2824delA (p.M942Cfs*21), and c.61G>T (p.E21*959) and c.2824delA (p.M942Cfs*21), respectively (Liu et al., 2022). None of these variants had been previously reported.

In a genetic study of induced abortions due to skeletal developmental abnormalities, whole exome sequencing identified a *de novo* missense variant in *NEK9* c.1973G>A (Li J. et al., 2025). This finding suggests that NEK9 dysfunction is directly implicated in human skeletal developmental malformations. Additionally, NEK9 plays important roles in osteoblast differentiation and bone formation. Its expression inhibition significantly impairs bone matrix mineralization, leading to skeletal maturation defects (Brum et al., 2017). Notably, mutations in NUP214, a nucleoporin that interacts with NEK9, also cause fetal hydrops and multiple joint contractures (Tamhankar et al., 2024). This observation highlights the importance of related pathways in joint development. Therefore, the severe skeletal phenotypes resulting from NEK9 mutations likely arise from the synergistic disruption of multiple cellular processes.

This phenotypic overlap is not coincidental; rather, it reflects the potentially irreplaceable and interconnected roles that NEK kinase family members play in regulating fundamental cellular processes during embryonic development, including cytoskeletal dynamics, ciliogenesis, and cell cycle control. Similar to NEK1, the extreme phenotypes caused by NEK9 mutations, such as lethal arthrogryposis, reveal that joint morphogenesis exhibits high sensitivity to NEK kinase function. Normal joint formation depends on precise cell proliferation, differentiation, and programmed cell death. Any factor that disrupts these processes may lead to joint fixation. NEK9 not only functions as a critical regulator of cell cycle progression but may also influence cell behavior through mechanisms such as regulating centrosome/spindle organization or directly participating in cilia assembly; its regulation of cell proliferation is particularly important in the context of p53 deficiency (Kurioka et al., 2014). During critical windows of limb bud development, even minor perturbations of NEK9 function may be amplified, resulting in aberrant mesenchymal cell behavior and ultimately causing irreversible joint contractures and osteogenic impairment.

NEK9-mediated phosphorylation of the essential light chain (ELC) of myosin represents an evolutionarily conserved, calcium-dependent mechanism for regulating muscle contraction (Muller et al., 2022). Mutations in the NEK9 gene disrupt this system, thereby impairing cardiac contraction and inotropy and predisposing to myocardial disease. This mechanism may also constitute a direct cause of the skeletal disorders characterized by multiple joint contractures (Muller et al., 2022). This defect may represent the crucial pathological basis underlying the skeletal developmental abnormalities associated with NEK9 variants. These findings not only expand the genetic spectrum of congenital arthrogryposis, but also provide critical evidence for understanding how NEK9 participates in skeletal developmental regulation. They underscore the indispensable role of this kinase in maintaining homeostasis of the joint and skeletal systems.

### 3.3 Common cellular phenotypes: coexistence of cell cycle arrest and ciliary defects

Dermal fibroblasts from patients serve as critical *in vitro* models for investigating the molecular mechanisms of NEK1- and NEK9-related disorders. Patient-derived fibroblasts harboring pathogenic mutations in either NEK1 or NEK9 exhibit markedly reduced proliferative capacity. This defect is intimately associated with impaired G2/M phase transition (Fry et al., 2012; O'Regan et al., 2007). Specifically, NEK1 functions as a centrosome-associated kinase. Loss of NEK1 function disrupts centrosome maturation and spindle assembly, thereby preventing cell entry into mitosis (Melo-Hanchuk et al., 2017; Mahjoub et al., 2005). NEK9 activates the spindle assembly checkpoint through phosphorylating NEK6 and NEK7, and mutations that abolish its kinase activity cause G2/M arrest and proliferative stagnation (Fry et al., 2012; van de Kooij et al., 2019; Bertran et al., 2011; Cullati et al., 2017; Belham et al., 2003). This cell cycle defect is primary and arises directly from the loss of core mitotic regulatory functions of NEK kinases.

A more critical phenotype is the widespread impairment of primary cilium assembly in cells with NEK1 or NEK9 mutations. However, existing evidences suggested that this defect is closely related to cell cycle arrest, but the causal direction may not be unidirectional. Primary cilium assembly depends strictly on cell cycle exit and entry into G0 phase (Kim and Tsiokas, 2011; Goto et al., 2017; Phua et al., 2017; Senatore et al., 2022). NEK1- or NEK9-mutant cells are arrested in G2/M phase and cannot complete mitosis to enter quiescence, thus losing the critical timing for initiating ciliogenesis (Kim and Tsiokas, 2011; Goto et al., 2017; Phua et al., 2017; Senatore et al., 2022). Ciliary number is significantly reduced or completely absent in mutant cells. Yet when cell cycle exit is forcibly induced through chemical means such as CDK inhibitors or serum deprivation, partial ciliary assembly capacity can be restored in a subset of cells (Casey et al., 2016; Youn et al., 2022; Monroe et al., 2016). These observations suggest that cell cycle status is an important factor regulating ciliogenesis; however, given that NEK kinases may also directly participate in cilia assembly, cell cycle arrest and ciliary defects in these diseases may constitute a mutually reinforcing pathological loop rather than a unidirectional cause-and-effect relationship.

Therefore, the core pathogenic mechanism of NEK1 and NEK9 LOF involve the combined effects of dysregulated cell cycle control and impaired ciliary function, which are tightly intertwined such that it is difficult to unequivocally distinguish cause from effect. Chondrocytes in the skeletal growth plate must undergo precise programs of proliferation, exit, and differentiation. NEK mutations cause cell cycle progression to stall in the proliferative phase without exit. This simultaneously reduces the number of chondrocytes available to enter hypertrophic and calcification stages and blocks cilia-mediated osteogenic differentiation signals such as Hedgehog and Wnt. Additionally, this may be compounded by abnormal muscle tone in joint regions. These converging mechanisms ultimately lead to skeletal phenotypes including shortened long bones, thoracic narrowing, and joint contractures (Casey et al., 2016; Thiel et al., 2011; Fry et al., 2012; Folahan and Barabutis, 2025; Costantini et al., 2023; Guasto and Cormier-Daire, 2021; Muller et al., 2022).

## 4 Integrative mechanisms of NEK kinases in skeletal development

### 4.1 Primary cell cycle dysregulation: the factor for proliferative failure in the epiphyseal growth plate

**FIGURE 2:**
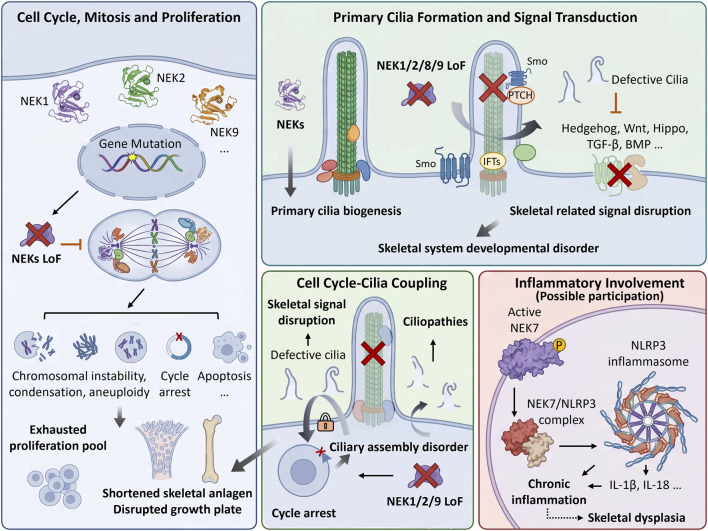
Pathogenic mechanisms by which LOF mutations of NEKs lead to skeletal dysplasia and chronic inflammation. LOF mutations of NEKs primarily trigger two major cellular events: (1) abnormalities in cell cycle, mitosis, and proliferation, characterized by chromosomal instability, impaired condensation, aneuploidy, and cell cycle arrest, which subsequently lead to exhaustion of the proliferative cell pool, disruption of the growth plate, and shortened skeletal elements (typically caused by variants in members such as NEK1, NEK2, and NEK9); (2) defects in primary cilia formation and associated signal transduction, including impaired ciliogenesis and dysregulation of signaling pathways such as Hedgehog, Wnt, Hippo, TGF-β, and BMP, collectively contributing to ciliopathies and skeletal system developmental disorders (exemplified by mutations in NEK1, NEK2, NEK8 and NEK9). Moreover, the cell cycle and ciliogenesis processes in which NEKs participate exhibit synergistic and interlocked regulation, jointly influencing skeletal formation (NEK1, NEK2 and NEK9 have been reported to be involved in this process). In addition, NEK7 can bind to and promote NLRP3 inflammasome activation, leading to the production of inflammatory cytokines such as IL-1β and IL-18, which trigger chronic inflammation and may also possibly participate in the pathological process of skeletal dysplasia.

Rapid and orderly proliferation of growth plate chondrocytes constitutes the core of longitudinal bone growth. This process depends on normal cell cycle operation, in which the NEK kinase family plays critical roles. The growth plate serves as the engine of longitudinal skeletal growth. The columnar arrangement of chondrocytes and their staged proliferation and differentiation provide the foundation for linear skeletal extension (Rosello-Diez and Joyner, 2015; Putz and Milz, 2016; Nilsson and Baron, 2004). Dysfunction of the NEK kinase family causes cells to fail in passing cell cycle checkpoints and initiating subsequent differentiation programs. These results in reduced cell numbers in the proliferative pool, representing the initiating event for the aforementioned structural developmental abnormalities (Figure 2) (Folahan and Barabutis, 2025; Mase et al., 2025). At the tissue level, cell proliferation defects caused by loss of NEK kinase function may manifest directly as disorganized growth plate cell arrangement and shortened skeletal anlagen. These changes lead to arrested skeletal development. However, this hypothesis awaits further validation through animal experimentation.

Loss of NEK kinase function leads to abnormal mitotic spindles that not only cause cell cycle arrest but also induce chromosomal structural instability. This further exacerbates cellular dysfunction and ultimately triggers apoptosis (Folahan and Barabutis, 2025; Peres de Oliveira et al., 2020). Sustained activation of the spindle assembly checkpoint can temporarily prevent erroneous segregation. However, prolonged arrest activates cell death pathways. Incorrect spindle microtubule-kinetochore attachments result in chromosome missegregation and aneuploidy (Weaver and Cleveland, 2005; Gregan et al., 2011). The high proliferative activity of growth plate chondrocytes renders their genomes particularly susceptible to endogenous stress. Loss of function in NEK1, NEK9, or NEK11 may impair cellular capacity to respond to replication stress or oxidative damage. This allows the accumulation of chromosomally unstable cells, which are subsequently eliminated through apoptosis or senescence pathways. The resulting further reduction in functional chondrocyte numbers creates a vicious cycle of proliferative failure and arrested skeletal development (Peres de Oliveira et al., 2020).

### 4.2 Ciliary dysfunction coupled with cell cycle defects

Ciliary structure and function are highly conserved throughout evolution. Mechanisms elucidated in lower organisms can typically be extrapolated directly to physiological and pathological processes in humans (Poprzeczko et al., 2019; Kim et al., 2010; Bisgrove and Yost, 2006). NEK2, NEK6, NEK7, and NEK9 are implicated in regulating axonemal microtubule formation in cilia and flagella of non-dividing cells (Fry et al., 2012; O'Regan et al., 2007; Baker et al., 2025). Given the tight coupling between cell cycle and ciliogenesis, ciliary defects resulting from NEK mutations are closely associated with cell cycle arrest. However, given that NEK kinase family members may also play direct regulatory roles in cilia assembly and maintenance, the causal relationship between the two may not be unidirectional linear but rather constitutes an intertwined pathological network. Cell cycle defects can disrupt the spatiotemporal dynamics of skeletal developmental signaling pathways (Fry et al., 2012; Kim and Tsiokas, 2011). Cilia function as signaling hubs on the cell surface that integrate multiple critical pathways during skeletal development. Their dysfunction can lead to global imbalance of the signaling network (Figure 2) (Guasto and Cormier-Daire, 2021).

Ciliary defects disrupt skeletal developmental signaling pathways most prominently through Hedgehog signaling (Huber and Cormier-Daire, 2012). Hedgehog signal transduction depends strictly on normal ciliary architecture, as cilia provide the localization and activation platform for key pathway components including Smoothened (Smo) and Gli transcription factors (De Mori et al., 2017). Conditional knockout of IFT80 in osteoprogenitor cells blocks canonical Hedgehog-Gli signal transduction through interfering with Smo ciliary localization, resulting in growth retardation and reduced bone mass (Yuan et al., 2016). Abnormal Ihh signaling during endochondral bone formation causes limb growth defects (Haycraft et al., 2007). Conditional ablation of IFT88 in limb bud mesenchymal cells not only causes polydactyly associated with aberrant Shh signaling but also induces limb axis shortening and abnormal endochondral ossification through disrupting Ihh signaling. This observation additionally suggests the involvement of other mechanisms (Haycraft et al., 2007).

In the context of NEK1 or NEK9 mutations, Hedgehog signal transduction blockade caused by ciliary dysfunction carries unique pathological significance. On one hand, cells accumulate in the proliferative zone due to cycle arrest and cannot receive differentiation signals transmitted through cilia. On the other hand, even the small fraction of cells that successfully exit the cycle may possess structurally or functionally abnormal cilia due to impaired NEK kinase functions in microtubule organization and cilia assembly. This leads to inefficient signal transduction. This dual blockade effect on both proliferation and differentiation likely accounts for the particularly severe phenotypes observed in NEK-associated skeletal disorders. Notably, although current evidence does not yet support NEK1 or NEK9 directly serving as components of ciliary structural proteins, their precise roles in regulating cilia assembly remain to be elucidated. The significant reduction in ciliary number is closely associated with cell cycle exit. However, the residual cilia that do form in a small fraction of cells may still be structurally or functionally aberrant due to loss of NEK1/9 function, thereby exacerbating the signaling deficits. Therefore, this mechanism has a different pathological trajectory from classical primary ciliopathies.

Beyond the Hedgehog pathway, ciliary defects may also broadly affect other signaling pathways critical for skeletal and articular development, among which the Wnt/planar cell polarity (PCP) pathway warrants particular attention (Quadri and Upadhyai, 2023). The Wnt/PCP pathway participates in regulating cell polarity and directional migration, processes essential for joint space formation, joint morphogenesis, and proper columnar arrangement of growth plate chondrocytes (Guasto and Cormier-Daire, 2021; Dreyer et al., 2022; Henderson et al., 2018). It must be emphasized that the effects of NEK1 or NEK9 mutations on these pathways are closely associated with ciliary dysfunction. Whether these kinases directly participate in signal transduction through their catalytic activity awaits further in-depth investigation.

### 4.3 A two-pronged approach: synergistic and interlocked regulation of the cell cycle and ciliogenesis

A precise and dynamic relationship of coordination and interlocking control exists between the cell cycle and ciliogenesis. This relationship is essential for maintaining cellular homeostasis and normal development. Primary cilium assembly is tightly coupled to exit from G0/G1 phases of the cell cycle, while ciliary disassembly constitutes a prerequisite for cell re-entry into the proliferative cycle (Kim and Tsiokas, 2011). The cilium itself may function as a physical checkpoint, with its presence or absence directly influencing cell cycle re-entry progression (Kim and Tsiokas, 2011). Following serum starvation-induced quiescence, cells initiate primary cilium assembly. Upon serum re-addition to stimulate cell cycle re-entry, cilia are rapidly resorbed (Kim and Lee, 2017).

The molecular basis of this regulation involves multiple layers including centrosomal protein conversion, periodic changes in kinase activity, and dynamic membrane remodeling (Ishikawa and Marshall, 2011; Joukov and De Nicolo, 2019; Seeley and Nachury, 2010). The centrosomal protein Nde1 has been identified as a negative regulator of ciliary length. Its absence causes ciliary elongation and delays cell cycle re-entry (Kim et al., 2011). Additionally, phosphoinositide signaling plays a critical role in the ciliary tip excision process, forming a molecular bridge between the ciliary life cycle and the cell division cycle (Phua et al., 2017).

The NEK kinase family serves as a critical node linking the regulatory networks of cell cycle progression and primary cilium assembly-disassembly (Meirelles et al., 2014; Li YY. et al., 2021). Residual cilia resulting from NEK2 knockout promote asymmetric inheritance of ciliary signaling components and ciliary reassembly following cell division (Viol et al., 2020). Studies have demonstrated that NEK2 promotes microtubule disassembly of cilia through phosphorylating and activating the kinesin Kif24, thereby ensuring effective ciliary disassembly before cells enter mitosis. This mechanism operates independently of Aurora A, and functional interplay exists between these two kinases in maintaining the dynamic equilibrium of ciliogenesis regulation (DeVaul et al., 2017; Kim et al., 2015). Additionally, other NEK members such as NEK1 have been found to interact with Cep104, a protein associated with cilium assembly, suggesting their involvement in regulating ciliogenesis (Al-Jassar et al., 2017).

Therefore, normal function of NEK kinases safeguards the dynamic balance between proliferative states and cilia-dependent signal sensing (Peres de Oliveira et al., 2020; Meirelles et al., 2014; Li YY. et al., 2021). Given that NEK family members may play roles in both cell cycle regulation and cilia assembly, loss of their activity can simultaneously disrupt the equilibrium between cell cycle progression and ciliary signal feedback, with the two being tightly intertwined and mutually reinforcing, resulting in dual dysregulation of skeletal development at both proliferative control and morphogenetic signal interpretation levels (Figure 2). During skeletal development, the NEK family ensures coordinated linear growth and morphogenesis through synergistic regulation of cell cycle checkpoints and ciliary architecture.

### 4.4 Potential involvement of inflammation and stress response pathways

It should be emphasized that there is no direct evidence indicates that loss of NEK1 or NEK9 function activates the inflammasome pathway currently. Based on the established scaffolding role of NEK7 in inflammation regulation, which is independent of its catalytic activity, we explored the possibility that NEK1/NEK9 loss-of-function may indirectly affect inflammatory signaling through NEK7; however, the precise intersection point between these pathways remains unclear. Under normal conditions, NEK7 maintains low activity, whereas its expression becomes aberrantly elevated under pathological conditions, playing critical roles in the progression of multiple tumors and chronic inflammatory diseases (Wang et al., 2022). NEK7 has been demonstrated to directly interact with NLRP3 and serves as an essential kinase for NLRP3 inflammasome activation (He et al., 2016; Luo et al., 2025; Sharif et al., 2019). Additionally, the NLRP3 inflammasome has also been widely reported to be involved in the developmental process of the skeletal system and the maintenance of skeletal homeostasis (Zhou et al., 2022; Yang et al., 2024; Liu et al., 2024; Bai et al., 2026; Xu B. et al., 2025).

The NLRP3 inflammasome constitutes a key component of the innate immune system. Its activation promotes maturation and release of proinflammatory cytokines IL-1β and IL-18, thereby driving robust inflammatory responses (Sun et al., 2025). Conditional knockout of NEK7 in endothelial cells alleviates indoxyl sulfate-induced endothelial injury and NLRP3 inflammasome activation (Sun et al., 2025). In a sepsis-induced cardiomyopathy model, NEK7 inhibition improves cardiac function through suppressing NLRP3 inflammasome activation (Wen et al., 2025). Studies in rheumatoid arthritis have revealed that NEK7 directly binds NLRP3 and regulates its oligomerization and activation downstream of potassium efflux, thereby participating in synovial inflammation and bone destruction through NLRP3 inflammasome activation (He et al., 2016). Inhibition of NEK7 expression in macrophages attenuates hepatic inflammatory responses, suppresses NLRP3 inflammasome pathway activation, and prevents the initiation and progression of fibrotic lesions (Jin et al., 2026).

Potassium efflux triggers JNK1-mediated phosphorylation of NEK7 at Thr190 and 191, enhancing NEK7 binding to NLRP3 and thereby amplifying inflammasome activation (Xu J. et al., 2025). Conversely, PLK4-mediated phosphorylation of NEK7 at Ser204 attenuates NEK7-NLRP3 interaction, thereby suppressing inflammasome assembly (Yang et al., 2020). Additionally, PLK1-mediated phosphorylation of NEK7, potentially at Ser221 and Ser260, has also been shown to enhance NEK7-NLRP3 binding (Luo et al., 2025). Cysteine residues of NEK7 can undergo glutathionylation, and deglutathionylation by GSTO1-1 is required for NLRP3 activation (Hughes et al., 2019). These modifications collectively regulate the strength and timing of NEK7-NLRP3 interaction. Notably, the catalytic kinase activity of NEK7 is not required for its regulatory function on NLRP3. Its core role lies in serving as a structural scaffold. Although NEK6 shares high homology with NEK7 in the catalytic domain, NEK6 alone cannot activate NLRP3 (He et al., 2016). Studies have indicated that a single amino acid residue difference (R121 in NEK7 corresponding to Q132 in NEK6) determines their distinct capacities to bind NLRP3 and activate the inflammasome (Jeltema et al., 2022). In summary, given the lack of evidence for a direct link between NEK1/NEK9 loss-of-function and the NEK7-NLRP3 inflammatory pathway, as well as the unresolved nature of the potential intersection point between them, the hypothesis that inflammatory mechanisms contribute to NEK-related skeletal disorders awaits further validation in future studies.

## 5 Potential roles of other NEK family members in skeletal homeostasis: association-based inferences from molecular functions

### 5.1 NEK2: mitotic regulator and indirect associations with skeletal development

NEK2 localizes to centrosomes and forms complexes with protein phosphatase 1 (PP1) (Helps et al., 2000). The formation of this kinase-phosphatase complex is implicated in coordinating centrosome separation and cell cycle progression (Helps et al., 2000). NEK2 expression is significantly upregulated in various human malignancies, and its overexpression is closely associated with malignant proliferation and poor prognosis, establishing it as a promising new target in cancer therapeutics (Zhang et al., 2021; Yao et al., 2019; Xia et al., 2025). In studies of hepatocellular carcinoma, gastric cancer, non-small cell lung cancer, glioma, prostate cancer, and colorectal cancer, NEK2 overexpression has been demonstrated to promote cell proliferation, migration, and epithelial-mesenchymal transition through regulating β-catenin/Wnt and ERK/MAPK signaling pathways, ultimately leading to chromosomal instability (Lai et al., 2017; Gu et al., 2017; Neal et al., 2014; Zeng et al., 2015; Liu et al., 2017; Shah et al., 2022; Fan et al., 2019).

Longitudinal bone growth depends critically on rapid and orderly proliferation and differentiation of growth plate chondrocytes. This process requires precise cell cycle regulation and mitotic fidelity. NEK2 regulates timely centrosome separation during G2/M phase through phosphorylating centrosomal linker proteins including C-Nap1 and Cep68. This ensures proper spindle assembly and thereby guarantees equal chromosome segregation in rapidly dividing cells such as chondroprogenitors (Man et al., 2015). Theoretically, NEK2 regulation of centrosome separation through phosphorylating linker proteins is essential for maintaining genomic stability, and its dysfunction may cause mitotic defects. Gain-of-function mutations in NEK2 could lead to excessive cell proliferation and may associate with certain skeletal overgrowth disorders. Conversely, LOF mutations may cause mitotic defects, cell cycle arrest, or apoptosis, impairing growth plate function and resulting in skeletal growth retardation or malformation.

However, inferring skeletal developmental necessity from the importance of cell cycle regulation involves a logical leap. Functional redundancy may exist among NEK2, NEK1, and NEK9 in cell cycle control, and defects in a single gene may be compensated by other family members. Recent studies have revealed that NEK2 plays a detrimental role in chondrocyte degeneration during osteoarthritis. Its overexpression promotes extracellular matrix degradation and accelerates joint degeneration through stabilizing ATF2 and suppressing autophagy (Li Z. et al., 2025). This observation indicates that NEK2 function relates to pathological processes in the skeletal joint system, but does not support reverse inference of its necessity in physiological skeletal development.

It must be clarified that at the current level of evidence, incorporating NEK2 into core mechanistic discussions of NEK-related skeletal diseases lacks sufficient justification. Future research may construct mouse models with conditional knockout of NEK2 in chondrocytes or employ specific NEK2 inhibitors for *in vivo* intervention. Systematic investigation of effects on growth plate morphology, endochondral ossification, and long bone development will hopefully reveal the authentic roles of NEK2 in both physiological skeletal development and pathological skeletal malformation.

### 5.2 NEK6 and NEK7: downstream effectors of NEK9 and potential inflammatory modulators

Recent studies have revealed functions of NEK6 and NEK7 in non-mitotic processes. The specific role of NEK7 in NLRP3 inflammasome activation provides novel perspectives for understanding the functional diversity of the NEK family (He et al., 2016; Luo et al., 2025; Sharif et al., 2019). Beyond serving as potential molecular nodes connecting local articular stress microenvironments with pathological inflammatory responses, NEK6 and NEK7 participate in regulating endocytosis and intracellular membrane trafficking at the cellular level (Lazetic et al., 2018; Joseph et al., 2023). These processes directly influence cellular capacity to respond to internal and environmental changes (Lazetic et al., 2018; Joseph et al., 2023). Loss of NEK6 or NEK7 function causes missorting of mannose-6-phosphate receptors from endosomes and disrupts early and recycling endosomal compartments (Joseph et al., 2023). This results in excessive tubular structures in recycling endosomes and impairs endocytic transport (Joseph et al., 2023). In the skeletal system, homeostasis of osteoblasts, osteoclasts, and chondrocytes depends on precise cellular signal transduction and material transport. NEK6 and NEK7 may serve as bridging molecules between cellular stress responses and inflammasome activation through maintaining normal endomembrane system function. Their dysfunction could transform intracellular stress such as metabolic pressure into sustained inflammatory signal output.

During physiological skeletal development, signal transduction of key morphogens such as BMP and Wnt depends critically on endocytosis, recycling, and degradation of their receptors (Guasto and Cormier-Daire, 2021; Papathanasiou et al., 2012; Priestley et al., 2023). The nematode NEKL-3, an ortholog of NEK6 and NEK7, participates in regulating basolateral uptake of BMP receptors SMA-6 and DAF-4 in epidermal cells (Joseph et al., 2023). Under pathological conditions such as osteoarthritis, articular cartilage experiences sustained mechanical stress and metabolic pressure. Dysfunction of NEK6 or NEK7 may cause aberrant intracellular membrane trafficking in chondrocytes. This could lead to sustained activation of inflammatory cytokine receptor signaling or missorting and secretion of matrix-degrading enzyme precursors, thereby exacerbating destruction of the extracellular matrix scaffold (Dennis and Briggs, 2025). Concurrently, endosomal dysfunction may impair autophagic flux, resulting in defective clearance of damaged organelles and protein aggregates. This further aggravates cellular stress and promotes activation of inflammasomes including NLRP3, forming a vicious cycle of cartilage destruction and intensifying pathological damage to the skeletal system.

NEK6 and NEK7 function as downstream effectors of NEK9 in the canonical mitotic regulatory pathway, forming a functional cascade with NEK9 (Fry et al., 2012; van de Kooij et al., 2019; Bertran et al., 2011; Cullati et al., 2017; Belham et al., 2003). The binding of DYNLL/LC8 to NEK9 is regulated by phosphorylation levels at Ser944 of NEK9. This regulation is essential for controlling downstream signal transduction through the NEK6/7 module (Gallego et al., 2013). In NEK9-related skeletal developmental disorders, impaired function of NEK6 or NEK7 likely represents a downstream consequence of cell cycle arrest rather than an independent pathogenic factor. Spindle assembly defects caused by NEK9 mutations may propagate through NEK6/7 pathways. However, the absence of reports linking NEK6 or NEK7 mutations to human skeletal dysplasia or arthrogryposis syndromes renders independent discussion of their roles in skeletal development genetically unfounded.

Investigating NEK6 and NEK7 within the context of skeletal metabolism and inflammatory networks can enrich understanding of NEK family functions and provide potential therapeutic targets for skeletal and articular diseases. In-depth studies of NEK6 and NEK7 expression and function in skeletal development and joint diseases may reveal broader roles of the NEK family. It must be clarified that associations between these functions and NEK9-related skeletal diseases remain largely speculative. Whether NEK6 and NEK7 participate in skeletal cell signal transduction through regulating endocytosis and intracellular membrane trafficking currently lacks direct evidence. Similarly, whether the role of NEK7 in inflammasome activation modifies articular phenotypes of NEK9-related diseases requires further experimental validation.

## 6 Research models, translational directions, and clinical implications

### 6.1 Applications and limitations of current research models

**FIGURE 3:**
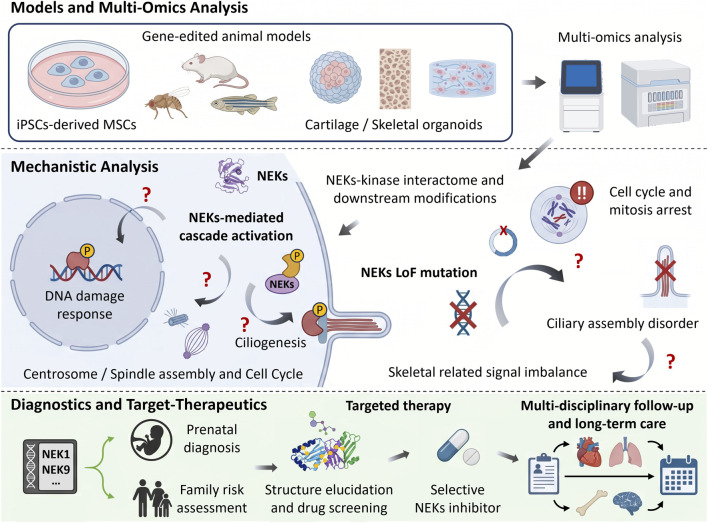
Proposed future research framework for investigating the molecular mechanisms underlying NEK-mediated skeletal disorders, as well as related clinical diagnosis, therapeutic intervention strategies. At the modeling level, approaches include patient iPSCs-derived MSCs with subsequent induction of osteogenic or chondrogenic differentiation, gene-edited animal models, and various forms of cartilage or skeletal organoids. In-depth mechanistic analysis integrates multi-omics with molecular biology studies. Current research has summarized the main biological roles of the downstream cascade reactions mediated by NEK kinases into three aspects: cell division and cell cycle regulation (mainly involving NEK1, NEK2, NEK3, NEK4, NEK5, NEK6, NEK9), cilia formation (mainly involving NEK1, NEK2, NEK4, NEK5, NEK8, NEK9, NEK10), and DNA damage response and repair (mainly involving NEK1, NEK4, NEK10, NEK11). After delineating the downstream cascade signals mediated by NEKs, further studies aim to elucidate the regulatory network through which NEKs loss-of-function mutations lead to cell cycle and mitotic arrest, impaired ciliogenesis and DNA damage response, ultimately resulting in skeletal dysplasia. Furthermore, for clinical diagnosis and intervention strategies, NEK1 and NEK9, among other members, should be prioritized as susceptibility genes for skeletal dysplasia. When facing skeletal dysplasia of unclear etiology, the priority of NEK testing should be raised. Based on the molecular regulatory networks involving these susceptibility genes, targeted drugs should be designed and developed, and long-term follow-up and mechanistic studies should be established in subsequent clinical practice.

Patient-derived induced pluripotent stem cells (iPSCs) provide a unique platform for modeling NEK-related skeletal developmental disorders in a human cellular context (Figure 3) (Kawai et al., 2019; Lee et al., 2015). The application of iPSC technology enables researchers to recapitulate disease molecular mechanisms *in vitro*, which holds significant importance for disease modeling and drug screening. However, iPSC models possess certain limitations. The *in vitro* culture environment cannot fully replicate the complex tissue microenvironment *in vivo*. Consequently, differentiated cell populations may exhibit heterogeneity under some circumstances, which partially restricts their capacity to reflect pathophysiological processes at the whole organ or organism level (Dakhore et al., 2018). Additionally, although iPSC technology has demonstrated potential in numerous fields, challenges remain in maintaining genomic stability and ensuring clinical safety (Vadodaria et al., 2020).

Genome-edited animal models, such as mice and zebrafish, serve as indispensable tools for investigating NEK gene function during organismal development. These models provide essential materials for exploring pathogenetic mechanisms, pathological processes, prevention, and treatment of human diseases (Li G. et al., 2022; Wang et al., 2015). The advantages of animal models include the capacity to conduct *in vivo* loss-of-function or gain-of-function studies and preclinical therapeutic testing. However, their limitations lie in potential interspecies differences, as gene function in model organisms cannot be fully equated with that in humans. Furthermore, the technical complexity, extended time requirements, and high costs associated with constructing tissue-specific or cell type-specific conditional knockout models restrict their broad application in research (Casey et al., 2016).

Organoid technologies, such as joint and growth plate organoids, provide a revolutionary platform for investigating skeletal morphogenetic defects caused by NEK mutations in three-dimensional environments that more closely approximate physiological conditions (Klompstra et al., 2025; Fan et al., 2025; Motoike et al., 2025; Zhang et al., 2024). Cartilage and bone tissue organoids based on various scaffold materials can form miniature skeletal tissues with specific spatial architectures and cellular diversity. These organoids recapitulate growth plate-like columnar proliferative patterns and simulate key processes including *in vivo* chondrocyte differentiation, apoptosis, and extracellular matrix deposition. They thereby better mimic *in vivo* skeletal development and homeostasis and facilitate revealing how NEK mutations disrupt three-dimensional skeletal morphogenesis, leading to malformations such as congenital arthrogryposis (Motoike et al., 2025; Zhu et al., 2026). However, this technology remains imperfect. Limitations particularly exist in vascularization and long-term stability, potentially preventing full simulation of late-stage disease changes. Furthermore, constructing phenotypically stable disease organoids remains challenging. Future research should integrate iPSCs, animal models, and organoids, combined with multi-omics analysis, to deeply resolve the functional networks of NEK kinases in skeletal diseases.

### 6.2 Future research directions

The NEK kinase family is frequently associated with both tumors and skeletal developmental abnormalities. Notably, the molecular mechanisms underlying these disease states differ fundamentally. In tumors, NEK family members typically exhibit overexpression or aberrant activation, consistent with their critical functions in cell cycle regulation, centrosome duplication, and mitotic progression. In contrast, skeletal developmental abnormalities or arthrogryposis are predominantly caused by LOF mutations that reduce protein expression or abolish catalytic activity (Casey et al., 2016; Thiel et al., 2011; Baker et al., 2025; Folahan and Barabutis, 2025). This dual pathogenic pattern of gain-of-function versus LOF suggests that NEK family members participate in disease pathogenesis through mechanism-specific modalities.

Currently, the pathogenic mechanisms of the NEK kinase family in skeletal development and congenital arthrogryposis syndromes remain incompletely defined, and the specific etiologies of numerous patients await elucidation (Quarmby and Mahjoub, 2005; Moniz et al., 2011). To deeply clarify the etiological mechanisms of such disorders, future research should systematically screen for novel pathogenic genes and mutation types within the NEK family in patient cohorts using whole exome or whole genome sequencing technologies (Scocchia et al., 2021). Concurrently, given the important roles of NEK kinases in regulating primary ciliary function, centriolar and mitotic processes, and DNA damage response, integrating multi-omics approaches to investigate their interacting proteins and downstream molecular modification patterns will enable more profound decryption of NEK kinase roles in these biological processes (Figure 3) (Baker et al., 2025; Meirelles et al., 2014).

Furthermore, to deeply resolve the molecular mechanisms by which NEK mutations regulate spatiotemporal dynamics of specific developmental signaling pathways, research should move beyond simple genotype-phenotype association paradigms and systematically investigate the underlying complex biological processes. A central question for subsequent research concerns the temporal sequence between cell cycle defects and ciliary defects initiated by NEK mutations during pathogenesis, or whether common pathogenic triggers exist. Based on these mechanistic studies, therapeutic strategies may be explored for patients with NEK mutations causing ciliary-Hedgehog signaling pathway abnormalities, such as utilizing small molecule drugs including Smo agonists or antagonists to modulate aberrant Hedgehog signaling activity and thereby rescue skeletal development (Baker et al., 2025).

Additionally, given that arthrogryposis is frequently accompanied by soft tissue fibrosis and chronic inflammation, testing antifibrotic agents such as pirfenidone or tissue-targeted anti-inflammatory drugs to evaluate their capacity to alleviate restricted joint mobility may serve as an adjunct therapeutic strategy for preclinical assessment (Medina et al., 2019; Zhou et al., 2016; Wells and Leung, 2020). From a long-term perspective, developing highly selective inhibitors or allosteric modulators targeting NEK kinase activity, or intervening in their downstream effector molecules, will also provide novel possibilities for targeted therapy of these disorders (Figure 3) (Li H. et al., 2025; Marzin and Cormier-Daire, 2020; Nguyen et al., 2023; Chen L. et al., 2025).

### 6.3 From basic mechanisms to novel insights into disease understanding

The core function of the NEK kinase family lies in regulating cell cycle progression. Loss of their function primarily causes mitotic arrest and blocks ciliogenesis. This creates a unique dual phenotype characterized by concurrent proliferative impairment and disrupted signal transduction. This mechanism is fundamentally distinct from primary ciliopathies caused by mutations in structural ciliary proteins alone (Zhao et al., 2021; De Jesus-Rojas et al., 2023; Kim and Lee, 2017).

In mammals, NEK family functions concentrate in three domains including centrosome and mitosis, ciliogenesis, and DNA damage response. Many members such as NEK1 and NEK8 participate in multiple domains simultaneously, thereby forming complex intersecting molecular regulatory networks. This property highlights their potential as connecting nodes between different functional networks within the cell. Such cross-connecting characteristics enable NEK kinases to precisely regulate relevant cellular events. Aberrations in these regulatory processes during specific tissue developmental programs consequently lead to disease pathogenesis (Fry et al., 2012; Meirelles et al., 2014; Li YY. et al., 2021; Moniz et al., 2011; Parker et al., 2007).

Dysfunction of the NEK family is closely associated with various diseases including cancer and ciliopathies (Baker et al., 2025). Therapeutic strategies for NEK-related skeletal disorders should target the primary defect in cell cycle regulation rather than focusing solely on ciliogenesis and ciliary dysfunction. Promoting cell cycle exit or alleviating mitotic checkpoint arrest may prove more effective in ameliorating disease phenotypes than merely rescuing ciliary signaling. Future in-depth dissection of NEK family interaction networks within specific developmental contexts will hopefully enable a deeper understanding of the complexity of human developmental disorders and identify novel entry points for intervention strategies.

### 6.4 Molecular diagnosis of NEK-related skeletal and articular diseases

In complex cases presenting with skeletal dysplasia accompanied by multiple joint contractures, molecular diagnosis serves as the key to identifying etiology and guiding clinical management (Lindelof et al., 2025; Semler et al., 2019). For fetuses or neonates presenting with severe lethal skeletal dysplasia accompanied by multiple joint contractures, incorporating NEK family members, particularly NEK1 and NEK9, into core candidate gene testing holds significant clinical importance for precise genetic counseling and effective prenatal intervention.

A prenatal study identified that among four cases suspected of skeletal dysplasia before 16 weeks of gestation, one fetus harboring NEK1 gene mutations was diagnosed with lethal skeletal abnormalities, highlighting the potential role of this gene in severe skeletal developmental disorders (Jelin et al., 2022). When probands carry variants associated with severe lethal phenotypes, the risk of recurrence in subsequent pregnancies for the same parents is 25% under autosomal recessive inheritance patterns (Jelin et al., 2022). This underscores the importance of prenatal diagnosis. For patients presenting with clinical phenotypes suggestive of ciliary dysfunction, the priority of NEK1 gene testing should be further elevated.

Therefore, mechanistic studies have established the importance of the NEK family in skeletal dysplasia-related disorders. Consequently, developing a targeted gene panel that includes NEK1 and NEK9 is crucial for the rapid and accurate diagnosis of these severe and rare congenital skeletal and articular diseases. For patients diagnosed with skeletal dysplasia caused by mutations in NEK kinase family genes, potential developmental or functional abnormalities in other organs may coexist. Thus, a multidisciplinary long-term follow-up and management protocol should be established. This protocol should incorporate regular assessments of skeletal growth, cardiopulmonary function, and neurodevelopment to enable early intervention and improve patient prognosis (Figure 3).

## 7 Conclusion

The NEK kinase family, represented by NEK1 and NEK9, serves as molecular hubs whose LOF simultaneously disrupts the two pillars of the skeletal growth plate: mitotic progression driving cell proliferation and primary cilia serving as key signaling platforms. This two-pronged pathogenic mechanism, particularly the disruption of developmental signals such as Hedgehog and Wnt, explains why NEK mutations cause severe skeletal and articular malformations with multi-system involvement. Meanwhile, the roles of other NEK family members in inflammation and stress responses suggest that local microenvironmental dysregulation may play a significant and non-negligible role in disease phenotype formation.

Current research has primarily relied on patient cells and animal models. Future studies may utilize three-dimensional organoids, conditional gene knockouts, and stem cell gene correction models to dissect NEK kinase family functions in three-dimensional space and along developmental timelines. This approach will clarify the division of labor and cooperative interactions among different members in specific cell types and developmental stages, hopefully revealing unknown compensatory mechanisms.

From a medical perspective, research of NEK kinases exemplifies the value of elucidating universal molecular regulatory principles through rare disease mechanisms. It advances precise diagnosis and genetic counseling for skeletal dysplasia-related syndromes and establishes foundations for developing novel therapeutics. Potential therapeutic targets now specifically direct toward actionable cellular processes, such as rescuing ciliary signaling defects, correcting mitotic abnormalities, or modulating local microenvironment.

Although clinical translation still faces numerous challenges, clarified molecular mechanisms have established clear pathway foundations for intervention strategies including small-molecule drug development and gene therapy. The ultimate goal aims to improve prognosis and quality of life for affected pediatric patients, translating basic scientific discoveries into clinical benefit for skeletal development-related diseases.
