# Supplementary material for: NIMA-related kinase family at the nexus of skeletal development and congenital arthrogryposis: coordinated regulation of cell cycle and ciliary dynamics
Source: Front Genet. 2026 Jul 13;17:1883963. doi: 10.3389/fgene.2026.1883963 (PMC13401987; doi:10.3389/fgene.2026.1883963)
Supplement: Supplementary file 1 [file Table1.docx]

**Supplementary Table 1. Functional Roles and Disease Associations of NEK9 Family Members: Cell Cycle, Ciliary Regulation, and Phenotypes**

| **NEKs** | **Cell Cycle Regulation** | **Ciliogenesis** | **Animal Model Data** | **Human Disease Associations** | **PMID** |
| --- | --- | --- | --- | --- | --- |
| NEK1 | Involved in the perception and repair of DNA strand breaks during G1-S and G2-M transitions;  DNA damage response (DDR), G2/M checkpoint control | Coordinates cell cycle with centrosome/cilia cycle, localizes to basal body, necessary for normal ciliogenesis;  The absence of NEK1 significantly reduces the number of cilia and alters their morphology | Spontaneous mutations in kat or kat2J (NEK1 deficiency/loss of function) lead to progressive polycystic kidney disease, anemia, male infertility, facial abnormalities, and retinal degeneration;  Knockout mice of NEK1 develop polycystic kidney disease | ?Orofaciodigital syndrome II;  {Amyotrophic lateral sclerosis, susceptibility to, 24};  Short-rib thoracic dysplasia 6 with or without polydactyly | 23132929, 22040655, 20501547, 21211617, 10618398 |
| NEK2 | Centrosome disjunction/separation(G2/M) (phosphorylates C-Nap1, Rootlet in);  A core component of the human centrosome, centrosome separation;  Located at the distal end of the mother centriole and promotes the depolymerization of cilia before mitosis | Connected to ciliogenesis;  Influences microtubule dynamics affecting primary cilia formation | NEK2-deficient mice exhibited significantly increased cryptorchidism incidence, decreased Leydig cell number, reduced testis/body weights, and elevated sperm malformations | ?Retinitis pigmentosa67;  High expression in multiple types of cancer | 23132929, 32211891, 22613497, 40386949 |
| NEK3 | Not primarily involved in canonical cell cycle regulation;  Contributes to prolactin-dependent signaling and proliferation;  Cytoskeleton organization | No direct documented role in ciliogenesis | Lack of systematically characterized phenotypic studies on NEK3 gene-engineered mice in the literature. | High expression in some tumors. | 23132929 |
| NEK4 | Not primarily a mitotic kinase; involved in DNA damage repair (DDR), cell cycle checkpoint control, apoptosis | Phosphorylating the core protein OFD1 of cilia and localizing it to the centriole/organizing center;  Directly participates in the assembly and stability regulation of primary cilia | No published study on the phenotypic characteristics of globally knockout mice with NEK4 gene;  Emotional behavior circadian rhythm abnormalities and synaptic structure changes in NEK4 transgenic mice with forebrain conditional overexpression | N/A | 38534317, 23132929, 39448584 |
| NEK5 | Centrosome integrity in interphase; contributes to centrosome cohesion;  Promotes centrosome linker disassembly in mitosis | Localizes to basal bodies in monociliated cells | Lack of systematic phenotypic studies on NEK5 knockout or overexpression transgenic mice throughout the body in the literature;  The silencing of NEK5 significantly inhibited tumor growth *in vivo* | N/A | 25963817, 34196902, 30675923 |
| NEK6 | Mitotic spindle formation (downstream of NEK9, with NEK2, NEK7, and NEK9);  Centrosome separation and maturation;  Metaphase/anaphase progression | No direct primary cilia role documented | In the models of STZ-induced diabetic cardiomyopathy and TAC-induced pressure-overload cardiac hypertrophy, the NEK6 knockout mice all exhibited more severe cardiac hypertrophy, fibrosis and cardiac dysfunction;  In various cancer xenograft models, knockdown or inhibition of NEK6 can significantly inhibit tumor growth and enhance chemotherapy sensitivity | High expression in multiple types of cancer | 23132929, 19414596, 36867206, 24763737, 27899381, 39256367 |
| NEK7 | Microtubule-based mitotic spindle formation (module with NEK6/NEK9);  Centrosome separation;  Might be required to recruit γ-tubulin to the poles | No direct primary cilia role documented | Silencing of NEK7 inhibits tumor growth in xenograft tumor models;  Knocking down NEK7 in cardiomyocytes of mice with transverse aortic constriction (TAC) significantly reduced myocardial hypertrophy and activation of the inflammasome. | The regulation of NLRP3 inflammasome is associated with inflammatory diseases | 23132929, 19414596, 26921196, 35223495, 41064864, 26814970 |
| NEK8 | Have key roles in cilia in post-mitotic cells | Primary cilium assembly/function;  localizes to proximal segment of primary cilia in collecting tubules/ducts;  Knockdown of NEK8 reduces the number of ciliated cells and disrupts the localization of cilia | Loss or knockdown of NEK8 primarily causes developmental defects, ciliary dysfunction, cystic kidney disease, and suppresses tumor growth；  Overexpression or specific mutations (notably jck) promote renal cyst formation, ciliary overgrowth, and accelerate various cancers | ?Neonophthisis9;  Polycystic kidney disease 8;  Renal-hepatic-pancreatic dysplasia 2 | 23132929, 18235101, 23274954, 25599650, 37100815, 30333866, 16928806, |
| NEK9 | Upstream activator of NEK6/NEK7;  Centrosome separation and maturation; spindle assembly;  Activated by PLK1 and regulates the early centrosome separation through NEK6/7 and Eg5 | The fibroblasts of the patient showed defects in cilia formation;  Acts as selective autophagy adaptor for MYH9 in primary cilia formation;  Mutation in LC3-interacting region impairs in vivo cilia formation in kidneys | NEK9 knockout leads to cardiomyopathy in zebrafish and embryonic lethality in mice, with LIR mutant mice showing defective renal cilia formation;  NEK9 knockdown inhibits p53-deficient tumor growth and gastric cancer metastasis | ?Arthrogryposis, Perthes disease, and upward gaze palsy;  Lethal congenital contracture syndrome 10;  Nevus comedonicus, somatic | 23132929, 26908619, 34078910, 36266340, 25131192, 33500736 |
| NEK10 | DNA damage response (DDR), G2/M checkpoint in response to UV irradiation;  Forms trimeric complex with MEK1/RAF1, activates ERK1/2 | Ciliogenesis, regulate the length and function of cilia | In mice, NEK10 knockdown alleviates renal fibrosis and tubular injury, and its knockout inhibits lung colonization of lung cancer cells;  In zebrafish embryos, the NEK10 inhibitor GeGe3 suppresses intersegmental angiogenesis. In C. elegans, mutation of the NEK10 homolog inhibits ciliary degeneration | Ciliary dyskinesia, primary, 44 | 23132929, 32414360, 40934128, 38683979, 33064774 |
| NEK11 | **G2/M checkpoint** (activated by Chk1 upon UV-induced DNA damage);  Phosphorylated by CHK1, and phosphorylates CDC25A promoting its degradation | No direct documented role in ciliogenesis | No published study on the phenotypic characteristics of globally knockout or over expressed mice with *NEK11* gene | High expression in some tumors | 23132929, 19734889 |
