# Supplementary material for: NIMA-related kinase family at the nexus of skeletal development and congenital arthrogryposis: coordinated regulation of cell cycle and ciliary dynamics
Source: Front Genet. 2026 Jul 13;17:1883963. doi: 10.3389/fgene.2026.1883963 (PMC13401987; doi:10.3389/fgene.2026.1883963)
Supplement: Supplementary file 2 [file Table2.docx]

**Supplementary Table 2. Summary of NEK1 and NEK9 Mutations and Associated Diseases**

| **Gene** | **Variant** | **Start** | **End** | **Ref_Transcript** | **HGVS** | **Altered amino acid levels** | **Phynotype** | **PMID** |
| --- | --- | --- | --- | --- | --- | --- | --- | --- |
| NEK1 | frameshift | 170327747 | 170327748 | NM_012224.4 | c.3289_3290delAT | del 2 bp  codon 1097 | Amyotrophic lateral sclerosis | 29149916 |
| NEK1 | frameshift | 170327765 | 170327765 | NM_012224.4 | c.3272delC | del 1 bp  codon 1091 | Amyotrophic lateral sclerosis | 29149916 |
| NEK1 | frameshift | 170384421 | 170384421 | NM_012224.4 | c.2476delC | del 1 bp  codon 826 | Amyotrophic lateral sclerosis | 28935222 |
| NEK1 | frameshift | 170482995 | 170482996 | NM_012224.4 | c.1127_1128delAA | del 2 bp  codon 376 | Amyotrophic lateral sclerosis | 32462798 |
| NEK1 | frameshift | 170476942 | 170476942 | NM_012224.4 | c.1491delT | del 1 bp  codon 497 | Amyotrophic lateral sclerosis | 32462798 |
| NEK1 | frameshift | 170359358 | 170359358 | NM_012224.4 | c.2556delA | del 1 bp  codon 852 | Amyotrophic lateral sclerosis | 32462798, 40225153 |
| NEK1 | frameshift | 170354711 | 170354712 | NM_012224.4 | c.2785_2786delGA | del 2 bp  codon 929 | Amyotrophic lateral sclerosis | 33445179 |
| NEK1 | noncoding | 170510664 | 170510667 | NM_012224.4 | c.397-2_398delAGAA | del 4 bp  non-coding DNA | Amyotrophic lateral sclerosis | 32920598 |
| NEK1 | frameshift | 170498115 | 170498115 | NM_012224.4 | c.984delA | del 1 bp  codon 328 | Skeletal dysplasia | 35587316 |
| NEK1 | frameshift | 170483039 | 170483042 | NM_012224.4 | c.1081_1084delGAAG | del 4 bp  codon 360 | Short rib-polydactyly syndrome, Majewski type | 35574990 |
| NEK1 | frameshift | 170498084 | 170498085 | NM_012224.4 | c.1014_1015delTA | del 2 bp  codon 338 | Short rib-polydactyly syndrome, Majewski type | 33879512 |
| NEK1 | frameshift | 170476913 | 170476914 | NM_012224.4 | c.1519_1520delTT | del 2 bp  codon 506 | Amyotrophic lateral sclerosis | 35495032 |
| NEK1 | frameshift | 170506597 | 170506597 | NM_012224.4 | c.710delA | del 1 bp  codon 237 | Amyotrophic lateral sclerosis | 34544842 |
| NEK1 | frameshift | 170459017 | 170459018 | NM_012224.4 | c.1607_1608delAG | del 2 bp  codon 536 | Amyotrophic lateral sclerosis | 34544842 |
| NEK1 | frameshift | 170322813 | 170322816 | NM_012224.4 | c.3486_3489delAGAA | del 4 bp  codon 1162 | Short rib thoracic dysplasia | 36964991 |
| NEK1 | frameshift | 170509805 | 170509805 | NM_012224.4 | c.546delT | del 1 bp  codon 182 | Amyotrophic lateral sclerosis | 37223130 |
| NEK1 | frameshift | 170483025 | 170483026 | NM_012224.4 | c.1097_1098delGA | del 2 bp  codon 366 | Amyotrophic lateral sclerosis | 37223130, 40225153 |
| NEK1 | frameshift | 170384480 | 170384480 | NM_012224.4 | c.2417delG | del 1 bp  codon 806 | Amyotrophic lateral sclerosis | 36443167 |
| NEK1 | frameshift | 170498223 | 170498223 | NM_012224.4 | c.876delA | del 1 bp  codon 292 | Amyotrophic lateral sclerosis | 36443167, 34544842 |
| NEK1 | frameshift | 170523238 | 170523241 | NM_012224.4 | c.132_135delAAGA | del 4 bp  codon 44 | Multiple system atrophy | 39152783 |
| NEK1 | inframe | 170384534 | 170384536 | NM_012224.4 | c.2361_2363delGGG | del 3 bp  codon 787 | Amyotrophic lateral sclerosis | 37952009 |
| NEK1 | frameshift | 170327785 | 170327785 | NM_012224.4 | c.3252delT | del 1 bp  codon 1084 | Amyotrophic lateral sclerosis | 40389989 |
| NEK1 | gross | 170345899 | 170345936 | NM_012224.4 | c.2906_2943del38 | deletion 38 bp, c.2906_2943 | Amyotrophic lateral sclerosis | 35047667, 39843441, 35616356 |
| NEK1 | frameshift | 170458984 | 170458985 | NM_012224.4 | c.1640dupA | ins 1 bp  codon 547 | Short rib-polydactyly syndrome, Majewski type | 21211617 |
| NEK1 | frameshift | 170428879 | 170428880 | NM_012224.4 | c.1813dupA | ins 1 bp  codon 605 | Amyotrophic lateral sclerosis | 32462798 |
| NEK1 | frameshift | 170398594 | 170398595 | NM_012224.4 | c.2109dupC | ins 1 bp  codon 704 | Short rib thoracic dysplasia | 36398383 |
| NEK1 | inframe | 170506667 | 170506668 | NM_012224.4 | c.634_639dupGTACTG | ins 6 bp  codon 214 | Retinitis pigmentosa, juvenile | 36341712 |
| NEK1 | frameshift | 170384425 | 170384426 | NM_012224.4 | c.2471dupC | ins 1 bp  codon 824 | Amyotrophic lateral sclerosis | 37952009, 40389989 |
| NEK1 | nonsense | 170511894 | 170511894 | NM_012224.4 | c.379C>T | Arg127Term | Short rib-polydactyly syndrome, Majewski type | 21211617, 31589614, 40225153, 36398383, 34426522, 37849306, 37223130, 25525159 |
| NEK1 | missense | 170510629 | 170510629 | NM_012224.4 | c.433G>A | Gly145Arg | Short rib-polydactyly syndrome, Majewski type | 22499340 |
| NEK1 | missense | 170506549 | 170506549 | NM_012224.4 | c.758T>C | Leu253Ser | Short rib-polydactyly syndrome, Majewski type | 22499340 |
| NEK1 | missense | 170509837 | 170509837 | NM_012224.4 | c.514C>T | Pro172Ser | Jeune asphyxiating thoracic dystrophy | 25492405 |
| NEK1 | missense | 170428888 | 170428888 | NM_012224.4 | c.1805G>A | Arg602His | Autism | 27525107 |
| NEK1 | missense | 170482701 | 170482701 | NM_012224.4 | c.1196A>C | Glu399Ala | Amyotrophic lateral sclerosis | 26945885 |
| NEK1 | missense | 170509809 | 170509809 | NM_012224.4 | c.542A>G | Asn181Ser | Amyotrophic lateral sclerosis | 26945885 |
| NEK1 | missense | 170400673 | 170400673 | NM_012224.4 | c.1936G>C | Gly646Arg | Amyotrophic lateral sclerosis | 28642336 |
| NEK1 | missense | 170502002 | 170502002 | NM_012224.4 | c.859C>G | Pro287Ala | Amyotrophic lateral sclerosis, phenotype modifier | 28709720, 37952009, 30755392, 37875969 |
| NEK1 | missense | 170482659 | 170482659 | NM_012224.4 | c.1238T>C | Leu413Pro | Amyotrophic lateral sclerosis, phenotype modifier | 28709720 |
| NEK1 | missense | 170398475 | 170398475 | NM_012224.4 | c.2150A>G | Asn717Ser | Amyotrophic lateral sclerosis | 29149916 |
| NEK1 | nonsense | 170477195 | 170477195 | NM_012224.4 | c.1318C>T | Arg440Term | Amyotrophic lateral sclerosis | 29149916, 34275688, 36550190 |
| NEK1 | missense | 170511914 | 170511914 | NM_012224.4 | c.359A>G | His120Arg | Amyotrophic lateral sclerosis | 29149916 |
| NEK1 | missense | 170511929 | 170511929 | NM_012224.4 | c.344C>T | Ala115Val | Amyotrophic lateral sclerosis | 29149916 |
| NEK1 | missense | 170384529 | 170384529 | NM_012224.4 | c.2368G>A | Val790Ile | Amyotrophic lateral sclerosis | 29149916 |
| NEK1 | nonsense | 170428238 | 170428238 | NM_012224.4 | c.1873C>T | Arg625Term | Amyotrophic lateral sclerosis | 29149916 |
| NEK1 | missense | 170477000 | 170477000 | NM_012224.4 | c.1433G>A | Gly478Glu | Amyotrophic lateral sclerosis | 29149916 |
| NEK1 | missense | 170498104 | 170498104 | NM_012224.4 | c.995A>C | Lys332Thr | Amyotrophic lateral sclerosis | 29149916 |
| NEK1 | missense | 170398386 | 170398386 | NM_012224.4 | c.2239C>G | His747Asp | Amyotrophic lateral sclerosis | 29149916 |
| NEK1 | missense | 170398593 | 170398593 | NM_012224.4 | c.2111A>G | Asn704Ser | Amyotrophic lateral sclerosis | 29149916 |
| NEK1 | missense | 170506547 | 170506547 | NM_012224.4 | c.760G>A | Glu254Lys | Amyotrophic lateral sclerosis | 29149916 |
| NEK1 | nonsense | 170345819 | 170345819 | NM_012224.4 | c.3023C>G | Ser1008Term | Amyotrophic lateral sclerosis | 26945885, 40225153, 35896380, 32409511, 28935222, 37849306, 28123176, 29068549 |
| NEK1 | missense | 170321393 | 170321393 | NM_012224.4 | c.3746A>C | Asp1249Ala | Axial spondyloarthritis dysplasia | 28123176 |
| NEK1 | missense | 170506613 | 170506613 | NM_012224.4 | c.694C>T | Arg232Cys | Amyotrophic lateral sclerosis/Frontotemporal dementia | 28935222 |
| NEK1 | nonsense | 170398497 | 170398497 | NM_012224.4 | c.2128C>T | Arg710Term | Amyotrophic lateral sclerosis | 30093141 |
| NEK1 | nonsense | 170398346 | 170398346 | NM_012224.4 | c.2279G>A | Trp760Term | Amyotrophic lateral sclerosis | 30093141, 35193472 |
| NEK1 | missense | 170321700 | 170321700 | NM_012224.4 | c.3602A>G | Lys1201Arg | Amyotrophic lateral sclerosis | 30093141, 37952009 |
| NEK1 | missense | 170345924 | 170345924 | NM_012224.4 | c.2918C>A | Ser973Tyr | Amyotrophic lateral sclerosis | 30093141 |
| NEK1 | missense | 170428268 | 170428268 | NM_012224.4 | c.1843C>G | Arg615Gly | Amyotrophic lateral sclerosis | 30093141 |
| NEK1 | missense | 170429475 | 170429475 | NM_012224.4 | c.1673T>C | Leu558Pro | Amyotrophic lateral sclerosis | 30093141 |
| NEK1 | missense | 170321430 | 170321430 | NM_012224.4 | c.3709C>T | His1237Tyr | Pulmonary arterial hypertension | 30029678, 33770234, 35896380, 34426522 |
| NEK1 | missense | 170458991 | 170458991 | NM_012224.4 | c.1634T>C | Met545Thr | Amyotrophic lateral sclerosis | 29650794, 37952009 |
| NEK1 | missense | 170477000 | 170477000 | NM_012224.4 | c.1433G>C | Gly478Ala | Amyotrophic lateral sclerosis | 29650794 |
| NEK1 | missense | 170511878 | 170511878 | NM_012224.4 | c.395A>G | Gln132Arg | Amyotrophic lateral sclerosis | 29650794 |
| NEK1 | missense | 170483347 | 170483347 | NM_012224.4 | c.1021G>A | Ala341Thr | Amyotrophic lateral sclerosis | 30755392, 28889094 |
| NEK1 | nonsense | 170428877 | 170428877 | NM_012224.4 | c.1816G>T | Glu606Term | Amyotrophic lateral sclerosis | 32579787, 36398383 |
| NEK1 | nonsense | 170321674 | 170321674 | NM_012224.4 | c.3628A>T | Lys1210Term | Amyotrophic lateral sclerosis | 32772750 |
| NEK1 | missense | 170523693 | 170523693 | NM_012224.4 | c.89A>G | Tyr30Cys | Autism spectrum disorder | 33374967 |
| NEK1 | missense | 170506621 | 170506621 | NM_012224.4 | c.686A>G | Tyr229Cys | Amyotrophic lateral sclerosis | 32462798 |
| NEK1 | missense | 170506534 | 170506534 | NM_012224.4 | c.773T>C | Ile258Thr | Amyotrophic lateral sclerosis | 32462798 |
| NEK1 | missense | 170506534 | 170506534 | NM_012224.4 | c.773T>G | Ile258Arg | Amyotrophic lateral sclerosis | 32462798 |
| NEK1 | missense | 170459039 | 170459039 | NM_012224.4 | c.1586A>G | Glu529Gly | Amyotrophic lateral sclerosis | 32462798 |
| NEK1 | missense | 170429410 | 170429410 | NM_012224.4 | c.1738C>T | Arg580Cys | Amyotrophic lateral sclerosis | 32462798 |
| NEK1 | missense | 170428197 | 170428197 | NM_012224.4 | c.1914G>C | Trp638Cys | Amyotrophic lateral sclerosis | 32462798 |
| NEK1 | missense | 170347316 | 170347316 | NM_012224.4 | c.2871A>C | Gln957His | Amyotrophic lateral sclerosis | 32462798 |
| NEK1 | synonymous | 170347373 | 170347373 | NM_012224.4 | c.2814G>A | Arg938Arg | Developmental disorder | 33057194, 35982159 |
| NEK1 | nonsense | 170345826 | 170345826 | NM_012224.4 | c.3016C>T | Gln1006Term | Amyotrophic lateral sclerosis | 33589474 |
| NEK1 | missense | 170506573 | 170506573 | NM_012224.4 | c.734A>C | Asp245Ala | Amyotrophic lateral sclerosis | 33589474 |
| NEK1 | missense | 170520286 | 170520286 | NM_012224.4 | c.277A>G | Asn93Asp | Odontochondrodysplasia-like | 34149817 |
| NEK1 | missense | 170476898 | 170476898 | NM_012224.4 | c.1535C>T | Ala512Val | Frontotemporal dementia / amyotrophic lateral sclerosis | 33770234 |
| NEK1 | initiation | 170523780 | 170523780 | NM_012224.4 | c.2T>C | Met1Thr | Amyotrophic lateral sclerosis | 33445179 |
| NEK1 | missense | 170458988 | 170458988 | NM_012224.4 | c.1637A>G | Gln546Arg | Amyotrophic lateral sclerosis | 33445179 |
| NEK1 | missense | 170523675 | 170523675 | NM_012224.4 | c.107A>G | Asn36Ser | Amyotrophic lateral sclerosis | 33414559 |
| NEK1 | nonsense | 170477150 | 170477150 | NM_012224.4 | c.1363C>T | Gln455Term | Amyotrophic lateral sclerosis | 32920598 |
| NEK1 | nonsense | 170327784 | 170327784 | NM_012224.4 | c.3253G>T | Glu1085Term | Skeletal dysplasia | 35587316 |
| NEK1 | missense | 170327820 | 170327820 | NM_012224.4 | c.3217C>T | Arg1073Cys | Skeletal dysplasia | 35587316, 33445179 |
| NEK1 | synonymous | 170520332 | 170520332 | NM_012224.4 | c.231C>T | Tyr77Tyr | Autism spectrum disorder | 35982160, 35982159 |
| NEK1 | missense | 170520273 | 170520273 | NM_012224.4 | c.290G>A | Gly97Asp | Amyotrophic lateral sclerosis | 34564799 |
| NEK1 | missense | 170400652 | 170400652 | NM_012224.4 | c.1957T>C | Ser653Pro | Amyotrophic lateral sclerosis | 34544842 |
| NEK1 | missense | 170345856 | 170345856 | NM_012224.4 | c.2986T>G | Leu996Val | Amyotrophic lateral sclerosis | 34544842 |
| NEK1 | missense | 170477230 | 170477230 | NM_012224.4 | c.1283G>T | Ser428Ile | Amyotrophic lateral sclerosis | 34544842 |
| NEK1 | missense | 170384477 | 170384477 | NM_012224.4 | c.2420C>T | Pro807Leu | Amyotrophic lateral sclerosis | 34544842, 37952009, 30528349 |
| NEK1 | missense | 170509800 | 170509800 | NM_012224.4 | c.551G>T | Ser184Ile | Amyotrophic lateral sclerosis | 34544842 |
| NEK1 | missense | 170501993 | 170501993 | NM_012224.4 | c.868G>C | Ala290Pro | Amyotrophic lateral sclerosis | 34544842 |
| NEK1 | missense | 170523210 | 170523210 | NM_012224.4 | c.163T>G | Leu55Val | Amyotrophic lateral sclerosis | 34544842 |
| NEK1 | missense | 170428268 | 170428268 | NM_012224.4 | c.1843C>T | Arg615Cys | Amyotrophic lateral sclerosis | 34544842 |
| NEK1 | missense | 170322820 | 170322820 | NM_012224.4 | c.3482A>G | Asn1161Ser | Amyotrophic lateral sclerosis | 34544842 |
| NEK1 | missense | 170511936 | 170511936 | NM_012224.4 | c.337T>C | Cys113Arg | Amyotrophic lateral sclerosis | 34544842 |
| NEK1 | missense | 170354790 | 170354790 | NM_012224.4 | c.2707C>G | Leu903Val | Amyotrophic lateral sclerosis | 34544842 |
| NEK1 | missense | 170498103 | 170498103 | NM_012224.4 | c.996G>T | Lys332Asn | Amyotrophic lateral sclerosis | 34544842 |
| NEK1 | missense | 170359315 | 170359315 | NM_012224.4 | c.2599G>C | Asp867His | Amyotrophic lateral sclerosis | 34544842 |
| NEK1 | missense | 170498140 | 170498140 | NM_012224.4 | c.959C>A | Ala320Glu | Amyotrophic lateral sclerosis | 34544842 |
| NEK1 | missense | 170520280 | 170520280 | NM_012224.4 | c.283C>G | Gln95Glu | Amyotrophic lateral sclerosis | 34544842 |
| NEK1 | missense | 170398624 | 170398624 | NM_012224.4 | c.2080G>C | Glu694Gln | Amyotrophic lateral sclerosis | 34544842 |
| NEK1 | nonsense | 170508733 | 170508733 | NM_012224.4 | c.583G>T | Glu195Term | Amyotrophic lateral sclerosis | 35047667 |
| NEK1 | nonsense | 170520259 | 170520259 | NM_012224.4 | c.304G>T | Glu102Term | Amyotrophic lateral sclerosis | 35426263, 37223130 |
| NEK1 | missense | 170511896 | 170511896 | NM_012224.4 | c.377A>T | His126Leu | Short-rib thoracic dysplasia | 36653407 |
| NEK1 | nonsense | 170477119 | 170477119 | NM_012224.4 | c.1394G>A | Trp465Term | Amyotrophic lateral sclerosis | 37223130 |
| NEK1 | missense | 170482887 | 170482887 | NM_012224.4 | c.1142T>A | Ile381Asn | Amyotrophic lateral sclerosis | 37223130 |
| NEK1 | nonsense | 170345735 | 170345735 | NM_012224.4 | c.3107C>G | Ser1036Term | Amyotrophic lateral sclerosis | 37223130, 39891418, 39843441 |
| NEK1 | missense | 170520323 | 170520323 | NM_012224.4 | c.240G>A | Met80Ile | Retinitis pigmentosa, juvenile | 36341712 |
| NEK1 | nonsense | 170523180 | 170523180 | NM_012224.4 | c.193C>T | Gln65Term | Amyotrophic lateral sclerosis | 36443167, 34544842 |
| NEK1 | nonsense | 170354729 | 170354729 | NM_012224.4 | c.2768G>A | Trp923Term | Amyotrophic lateral sclerosis | 36443167, 34544842 |
| NEK1 | missense | 170322833 | 170322833 | NM_012224.4 | c.3469G>C | Glu1157Gln | Amyotrophic lateral sclerosis | 38625400 |
| NEK1 | nonsense | 170506547 | 170506547 | NM_012224.4 | c.760G>T | Glu254Term | Amyotrophic lateral sclerosis | 39058450 |
| NEK1 | missense | 170509828 | 170509828 | NM_012224.4 | c.523T>C | Cys175Arg | Amyotrophic lateral sclerosis | 37952009 |
| NEK1 | missense | 170345801 | 170345801 | NM_012224.4 | c.3041C>T | Ala1014Val | Amyotrophic lateral sclerosis | 37952009 |
| NEK1 | missense | 170359320 | 170359320 | NM_012224.4 | c.2594T>C | Ile865Thr | Amyotrophic lateral sclerosis | 37952009 |
| NEK1 | missense | 170520291 | 170520291 | NM_012224.4 | c.272G>T | Arg91Leu | Amyotrophic lateral sclerosis | 37952009 |
| NEK1 | initiation | 170523781 | 170523781 | NM_012224.4 | c.1A>G | Met1Val | Amyotrophic lateral sclerosis | 40389989 |
| NEK1 | missense | 170520304 | 170520304 | NM_012224.4 | c.259G>T | Asp87Tyr | Amyotrophic lateral sclerosis | 40389989 |
| NEK1 | missense | 170520291 | 170520291 | NM_012224.4 | c.272G>A | Arg91Gln | Amyotrophic lateral sclerosis | 40389989 |
| NEK1 | missense | 170510617 | 170510617 | NM_012224.4 | c.445A>C | Ile149Leu | Amyotrophic lateral sclerosis | 40389989 |
| NEK1 | missense | 170498200 | 170498200 | NM_012224.4 | c.899T>C | Ile300Thr | Amyotrophic lateral sclerosis | 40389989 |
| NEK1 | missense | 170498191 | 170498191 | NM_012224.4 | c.908T>C | Met303Thr | Amyotrophic lateral sclerosis | 40389989 |
| NEK1 | missense | 170429409 | 170429409 | NM_012224.4 | c.1739G>A | Arg580His | Amyotrophic lateral sclerosis | 40389989 |
| NEK1 | missense | 170428267 | 170428267 | NM_012224.4 | c.1844G>A | Arg615His | Amyotrophic lateral sclerosis | 40389989 |
| NEK1 | missense | 170398357 | 170398357 | NM_012224.4 | c.2268T>A | Asp756Glu | Amyotrophic lateral sclerosis | 40389989 |
| NEK1 | missense | 170345875 | 170345875 | NM_012224.4 | c.2967G>T | Lys989Asn | Amyotrophic lateral sclerosis | 40389989 |
| NEK1 | canonical-splice | 170498232 | 170498232 | NM_012224.4 | c.869-2A>G | IVS10 as A-G -2 | Short rib-polydactyly syndrome, Majewski type | 21211617, 25525159 |
| NEK1 | canonical-splice | 170509887 | 170509887 | NM_012224.4 | c.465-1G>A | IVS6 as G-A -1 | Short rib-polydactyly syndrome, Majewski type | 22482978 |
| NEK1 | canonical-splice | 170511961 | 170511961 | NM_012224.4 | c.313-1A>G | IVS4 as A-G -1 | Short rib-polydactyly syndrome, Verma-Naumoff | 22795106 |
| NEK1 | canonical-splice | 170498231 | 170498231 | NM_012224.4 | c.869-1G>T | IVS10 as G-T -1 | Jeune asphyxiating thoracic dystrophy | 25492405, 40225153, 27455347, 36550190 |
| NEK1 | exonic-splice | 170510598 | 170510598 | NM_012224.4 | c.464G>C | Ser155Thr | Oral-facial-digital syndrome II | 27530628 |
| NEK1 | exonic-splice | 170482671 | 170482671 | NM_012224.4 | c.1226G>A | Trp409Term | Oral-facial-digital syndrome II | 27530628, 31589614 |
| NEK1 | canonical-splice | 170428944 | 170428944 | NM_012224.4 | c.1750-1G>A | IVS19 as G-A -1 | Amyotrophic lateral sclerosis | 28642336, 40225153 |
| NEK1 | canonical-splice | 170359412 | 170359412 | NM_012224.4 | c.2504-2A>G | IVS25 as A-G -2 | Amyotrophic lateral sclerosis | 30093141, 37952009, 32462798 |
| NEK1 | canonical-splice | 170458958 | 170458958 | NM_012224.4 | c.1665+2T>C | IVS18 ds T-C +2 | Amyotrophic lateral sclerosis | 33589474 |
| NEK1 | canonical-splice | 170508709 | 170508709 | NM_012224.4 | c.606+1G>A | IVS8 ds G-A +1 | Amyotrophic lateral sclerosis | 32920598 |
| NEK1 | canonical-splice | 170428864 | 170428864 | NM_012224.4 | c.1827+2T>C | IVS20 ds T-C +2 | Amyotrophic lateral sclerosis | 37223130 |
| NEK1 | canonical-splice | 170322802 | 170322802 | NM_012224.4 | c.3499+1G>A | IVS31 ds G-A +1 | Amyotrophic lateral sclerosis | 36443167, 34544842 |
| NEK1 | canonical-splice | 170482706 | 170482706 | NM_012224.4 | c.1192-1G>T | IVS14 as G-T -1 | Amyotrophic lateral sclerosis | 36443167 |
| NEK1 | canonical-splice | 170315675 | 170315675 | NM_012224.4 | c.3764-1G>C | IVS33 as G-C -1 | Amyotrophic lateral sclerosis | 36443167 |
| NEK1 | canonical-splice | 170321671 | 170321671 | NM_012224.4 | c.3630+1G>T | IVS32 ds G-T +1 | Amyotrophic lateral sclerosis | 36443167 |
| NEK1 | splice | 170321514 | 170321514 | NM_012224.4 | c.3631-6G>A | IVS32 as G-A -6 | Amyotrophic lateral sclerosis | 37849306 |
| NEK1 | splice | 170510588 | 170510588 | NM_012224.4 | c.464+10A>G | IVS6 ds A-G +10 | Amyotrophic lateral sclerosis | 37952009 |
| NEK1 | exonic-splice | 170511877 | 170511877 | NM_012224.4 | c.396G>A | Gln132Gln | Amyotrophic lateral sclerosis | 40389989 |
| NEK1 | splice | 170510590 | 170510590 | NM_012224.4 | c.464+8G>T | IVS6 ds G-T +8 | Amyotrophic lateral sclerosis | 40389989 |
| NEK1 | splice | 170321812 | 170321812 | NM_012224.4 | c.3500-10T>A | IVS31 as T-A -10 | Short rib-polydactyly syndrome, lethal | 40147672 |
| NEK1 | frameshift | 170347340 | 170347340 | NM_012224.4 | c.2847delTinsGG | del 1 bp / ins 2 bp codon 949 | Short rib-polydactyly syndrome, Majewski type | 22499340 |
| NEK1 | frameshift | 170347340 | 170347340 | NM_012224.4 | c.2847delTinsGG | del 1 bp / ins 2 bp codon 949 | Short rib-polydactyly syndrome, Majewski type | 22499340 |
| NEK9 | frameshift | 75573235 | 75573235 | NM_033116.6 | c.1498delG | del 1 bp codon 500 | Lethal contracture syndrome | 29096039 |
| NEK9 | inframe | 75590815 | 75590817 | NM_033116.6 | c.329_331delACA | del 3 bp codon 110 | Short long bones, bowed femur, clubfeet, deviation of hand, scoliosis, micrognathia and abnormal filling stomach | 32333414, 38259611 |
| NEK9 | inframe | 75568443 | 75568445 | NM_033116.6 | c.1755_1757delAAC | del 3 bp codon 585 | Nevus comedonicus syndrome | 33502802 |
| NEK9 | frameshift | 75573301 | 75573301 | NM_033116.6 | c.1432delC | del 1 bp codon 478 | Fetal akinesia | 34740919 |
| NEK9 | frameshift | 75593508 | 75593509 | NM_033116.6 | c.115_116dupCG | ins 2 bp codon 39 | Lethal contracture syndrome | 35627109 |
| NEK9 | missense | 75563934 | 75563934 | NM_033116.6 | c.2042G>A | Arg681His | Joint contracture, limited upward gaze & Legg-Calve-Perthes disease | 26633546,39504961, 37644014 |
| NEK9 | nonsense | 75573244 | 75573244 | NM_033116.6 | c.1489C>T | Arg497Term | Skeletal dysplasia, lethal | 26908619 |
| NEK9 | missense | 75573228 | 75573228 | NM_033116.6 | c.1505A>G | Tyr502Cys | Cleft palate, proliferative retinopathy, and developmental delay | 30976112 |
| NEK9 | missense | 75555262 | 75555262 | NM_033116.6 | c.2525C>T | Thr842Ile | Two IUFD with congenital anomalies | 31130284 |
| NEK9 | missense | 75567826 | 75567826 | NM_033116.6 | c.1871A>G | Asn624Ser | Short long bones, bowed femur, clubfeet, deviation of hand, scoliosis, micrognathia and abnormal filling stomach | 32333414, 38259611 |
| NEK9 | missense | 75570567 | 75570567 | NM_033116.6 | c.1709T>A | Met570Lys | Developmental disorder | 33057194, 35982159 |
| NEK9 | missense | 75562101 | 75562101 | NM_033116.6 | c.2207A>G | Asn736Ser | Developmental disorder | 33057194, 35982159 |
| NEK9 | missense | 75570615 | 75570615 | NM_033116.6 | c.1661T>G | Leu554Arg | Developmental disorder | 33057194, 35982159 |
| NEK9 | nonsense | 75576537 | 75576537 | NM_033116.6 | c.1033C>T | Arg345Term | Lethal contracture syndrome | 33742171 |
| NEK9 | missense | 75570661 | 75570661 | NM_033116.6 | c.1615T>C | Cys539Arg | Lethal contracture syndrome | 35627109 |
| NEK9 | canonical-splice | 75580108 | 75580108 | NM_033116.6 | c.874-2A>G | IVS7 as A-G -2 | Fetal akinesia | 34740919 |
